# Drought‐responsive genes, late embryogenesis abundant group3 (*LEA3*) and vicinal oxygen chelate, function in lipid accumulation in *Brassica napus* and *Arabidopsis* mainly via enhancing photosynthetic efficiency and reducing ROS

**DOI:** 10.1111/pbi.13127

**Published:** 2019-04-26

**Authors:** Yu Liang, Kai Kang, Lu Gan, Shaobo Ning, Jinye Xiong, Shuyao Song, Lingzhi Xi, Senying Lai, Yongtai Yin, Jianwei Gu, Jun Xiang, Shisheng Li, Baoshan Wang, Maoteng Li

**Affiliations:** ^1^ Department of Biotechnology College of Life Science and Technology Huazhong University of Science and Technology Wuhan China; ^2^ Center for Plant Science Innovation and Department of Biochemistry University of Nebraska Lincoln Lincoln NE USA; ^3^ Hubei Research Institute of New Socialist Countryside Development Hubei Engineering University Xiaogan China; ^4^ Hubei Key Laboratory of Economic Forest Germplasm Improvement and Resources Comprehensive Utilization Hubei Collaborative Innovation Center for the Characteristic Resources Exploitation of Dabie Mountains Huanggang Normal University Huanggang China; ^5^ College of Life Science Shandong Normal University Jinan China

**Keywords:** *LEA3*, *VOC*, *Brassica napus*, drought tolerance, lipid accumulation, photosynthetic efficiency, reactive oxygen species

## Abstract

Drought is an abiotic stress that affects plant growth, and lipids are the main economic factor in the agricultural production of oil crops. However, the molecular mechanisms of drought response function in lipid metabolism remain little known. In this study, overexpression (OE) of different copies of the drought response genes *LEA3* and *VOC* enhanced both drought tolerance and oil content in *Brassica napus* and *Arabidopsis*. Meanwhile, seed size, membrane stability and seed weight were also improved in OE lines. In contrast, oil content and drought tolerance were decreased in the *AtLEA3* mutant (*atlea3*) and AtVOC‐RNAi of *Arabidopsis* and in both BnLEA‐RNAi and BnVOC‐RNAi *B. napus* RNAi lines. Hybrids between two lines with increased or reduced expression (LEA3‐OE with VOC‐OE,* atlea3* with AtVOC‐RNAi) showed corresponding stronger trends in drought tolerance and lipid metabolism. Comparative transcriptomic analysis revealed the mechanisms of drought response gene function in lipid accumulation and drought tolerance. Gene networks involved in fatty acid (FA) synthesis and FA degradation were up‐ and down‐regulated in OE lines, respectively. Key genes in the photosynthetic system and reactive oxygen species (ROS) metabolism were up‐regulated in OE lines and down‐regulated in *atlea3* and AtVOC‐RNAi lines, including *LACS9*, *LIPASE1*, *PSAN*, *LOX2* and *SOD1*. Further analysis of photosynthetic and ROS enzymatic activities confirmed that the drought response genes *LEA3* and *VOC* altered lipid accumulation mainly via enhancing photosynthetic efficiency and reducing ROS. The present study provides a novel way to improve lipid accumulation in plants, especially in oil production crops.

## Introduction

Drought is an important abiotic stress (Zhu, 2002) that can affect the morphological, physiological and biochemical characteristics of plants (Suzuki *et al*., 2014). With the loss of cellular homeostasis under drought, an accumulation of toxic metabolites injures plant cells (Miller *et al*., 2010; Xiong and Zhu, 2002). In addition, increased production of reactive oxygen species (ROS) could cause further damage to plant tissue (Miller *et al*., 2010).

Some progress has been made in understanding how plants respond to drought stress, and some drought‐responsive genes have been identified in plants (Zhang, 2015; Zhao *et al*., 2016). Significant drought stress can induce transcription of dehydration‐responsive element‐binding proteins (DREBs) in plants and then activate genes that are involved in detoxification, water and ion movement and chaperone functions, including LEA (Shinozaki and Yamaguchi‐Shinozaki, 2007; Wang *et al*., 2003; Xiong and Zhu, 2002; Yoshida *et al*., 2014). In this model, *LEA* genes are part of a category that could function in the protection of membranes and proteins. Previous studies have suggested that LEA‐type proteins could act as water‐binding molecules, playing important roles in macromolecule and membrane stabilization and in ion sequestration (Chakrabortee *et al*., 2012; Wang *et al*., 2003). Most LEA proteins are hydrophilic and are not thought to have a stable secondary structure (Bremer *et al*., 2017; Hincha and Thalhammer, 2012; Tunnacliffe *et al*., 2010). These structural characteristics are related to the prediction of their function in response to desiccation stress (Candat *et al*., 2014; Chakrabortee *et al*., 2007). VOC proteins are members of an enzyme superfamily with a common mechanistic attribute enabled by conserved active site residues. Glyoxalase I (GLYI) is a major member of the VOC family. It is a metalloenzyme that participates in the glyoxalase system, which has been reported to be a major pathway for the detoxification of methylglyoxal (MG) in living organisms. GLYI can use one molecule of glutathione (GSH) to convert MG to S‐D‐lactoylglutathione and functions in abiotic stress response (Mustafiz *et al*., 2014; Singla‐Pareek *et al*., 2003).

Lipids are essential for plants and humans; they provide not only the energy for metabolic processes but also a structural basis for cell membranes (Okazaki and Saito, 2014). In *Arabidopsis thaliana* and *Brassica napus*, oil is mainly stored in seeds as triacylglycerols (TAGs), together with starch and storage proteins (Graham, 2008). TAG biosynthesis is achieved through the coordinated action of multiple pathways in various subcellular compartments. The synthetic process could be briefly classified into different phases in plant cells (Chapman and Ohlrogge, 2012). Some genes in these TAG biosynthetic pathways were identified. Diacylglycerol acyltransferases (DGATs) are well known in TAG biosynthesis in plants. *DGAT1* is required for TAG accumulation in *Arabidopsis*; disruption of the *DGAT1* gene could result in a 20%–40% reduction in seed oil in *Arabidopsis*, while overexpression of *DGAT1* in vegetative tissues or seeds could lead to TAG accumulation (Yen *et al*., 2008; Zhang *et al*., 2009).

In plants, lipids also participate in signalling pathways that respond to environmental cues (Okazaki and Saito, 2014). In recent years, it was revealed that some enzymes involved in lipid metabolism could play a part in abiotic stress tolerance. For example, freezing tolerance was decreased with disruption of *AtDGAT1* (Arisz *et al*., 2018). A lipase, designated as HEAT INDUCIBLE LIPASE1 (HIL1), induces the catabolism of monogalactosyldiacylglycerol (MGDG) under heat stress in *Arabidopsis* leaves (Higashi *et al*., 2018). Whether abiotic stress response genes can also function in lipid metabolism remains unclear. In our previous study, it was revealed that the LEA and VOC proteins were highly expressed in *B. napus* plants that had high oil content (Gan *et al*., 2013). We also found that most of the *BnLEA* and *BnVOC* genes had higher expression levels under drought stress in *B. napus* with high oil content (Liang *et al*., 2016, 2017). These results suggested that drought‐stress‐responsive genes might have a relationship with lipid metabolism.

*Brassica napus* (AACC, 2*n* = 38) is an allopolyploid species with a triplicated genome structure and many duplicated genes (Chalhoub *et al*., 2014). Rapeseed is the third largest oilseed crop in the world, and many areas where it is planted are affected by drought. Thus, it is important to further study the exact roles of genes involved in oil accumulation and response to drought stress conditions. In the present study, oil content and drought resistance were analysed in *LEA3* and *VOC* transgenic and mutant plants in *Arabidopsi*s and rapeseed. RNA‐seq analysis revealed that FA synthesis was up‐regulated and FA degradation was down‐regulated in OE lines. In addition, in the photosynthetic system, chlorophyll synthesis was up‐regulated in OE lines and down‐regulated in *atlea3* and AtVOC‐RNAi. Hybrids between either OE lines or RNAi lines had corresponding effects on drought tolerance and oil content in *B. napus*. Finally, we confirmed that *LEA3* and *VOC* play an important role in lipid accumulation, mainly by enhancing photosynthetic efficiency and reducing ROS. The present research reveals that stress response genes could not only function in abiotic stress but also enhance oil accumulation in oil crops.

## Results

### *LEA3* and *VOC Arabidopsis* transgenic lines could enhance drought tolerance and lipid accumulation under drought treatment

To investigate the role of *LEA3s* and *VOC*s in oil metabolism, different copies of *BnLEA3* and *BnVOC* and the *AtLEA3* and *AtVOC* genes were cloned into plant expression vectors, and two kinds of vectors were constructed. One kind of vector contained the 35S CaMV promoter, and the other contained the glycinin promoter. These two vector types could be used to confer overexpression in whole plants and in only the seeds of transgenic lines, respectively (Figures S1, S2). These twenty OE vectors were all transformed into *Arabidopsis*. The red fluorescent protein (DsRed) gene was used to mark the transgenic seeds (Figure S1B).

**Figure 1:**
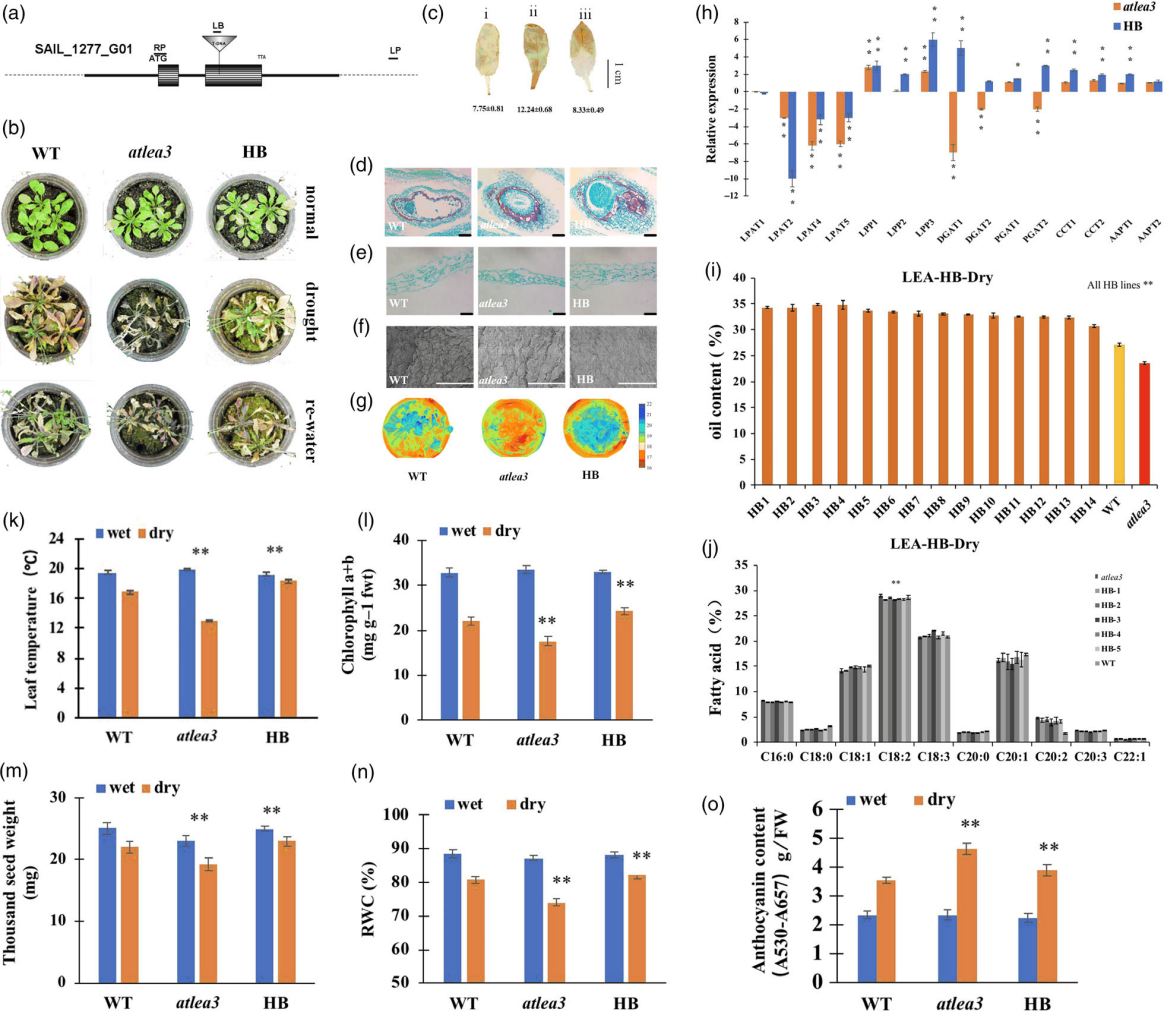
Drought tolerance and oil content of the *atlea3* mutant and complementation of the full‐length *AtLEA3* gene into *atlea3*. (a): Schematic diagram of the T‐DNA insertion mutant used in this study (*atlea3*). (b): Phenotype of the *atlea3* mutant, HB (complementary vector transformed into *atlea3*) and WT under normal, drought and rewatering conditions. (c): DAB staining of WT (i), *atlea3* (ii) and HB (iii). The number below indicates the quantitative data of H_2_O_2_ content, and unit is nmol/g FW. (d and e): Seed and leaf sections of WT,* atlea3* and HB under drought stress. Bar = 500 μm. (f): SEM observation of leaves of WT,* atlea3* and HB under drought conditions. Bar = 100 μm. (g): Images of mutant, HB and WT plants in a thermal imaging system under drought conditions. (h): Relative expression levels of genes in the TAG synthetic pathway in HB and *atlea3* under drought conditions. LPAT: lysophosphatidic acid acyltransferase, LPP: lipid phosphate phosphatase, DGAT: diacylglycerol acyltransferase, PDAT: phospholipid:diacylglycerol acyl transferase, CCT: choline‐phosphate:CTP cytidylyltransferase, AAPT: aminoalcohol‐phosphotransferase. (i): Oil contents of the HB lines and mutants under drought conditions. (j): FA compositions of the HB lines and mutants under drought conditions. (k–o): Leaf temperatures, chlorophyll a + b contents, RWCs, TSWs and anthocyanin contents of the HB lines and mutants under drought conditions. All the results are represented as the mean ± standard deviation (STD;* n* = 3). Statistically significant differences were determined using a two‐tailed paired Student's *t*‐test compared with WT plants under similar conditions, and the results are indicated by **P < 0.01 and *P < 0.05.

**Figure 2:**
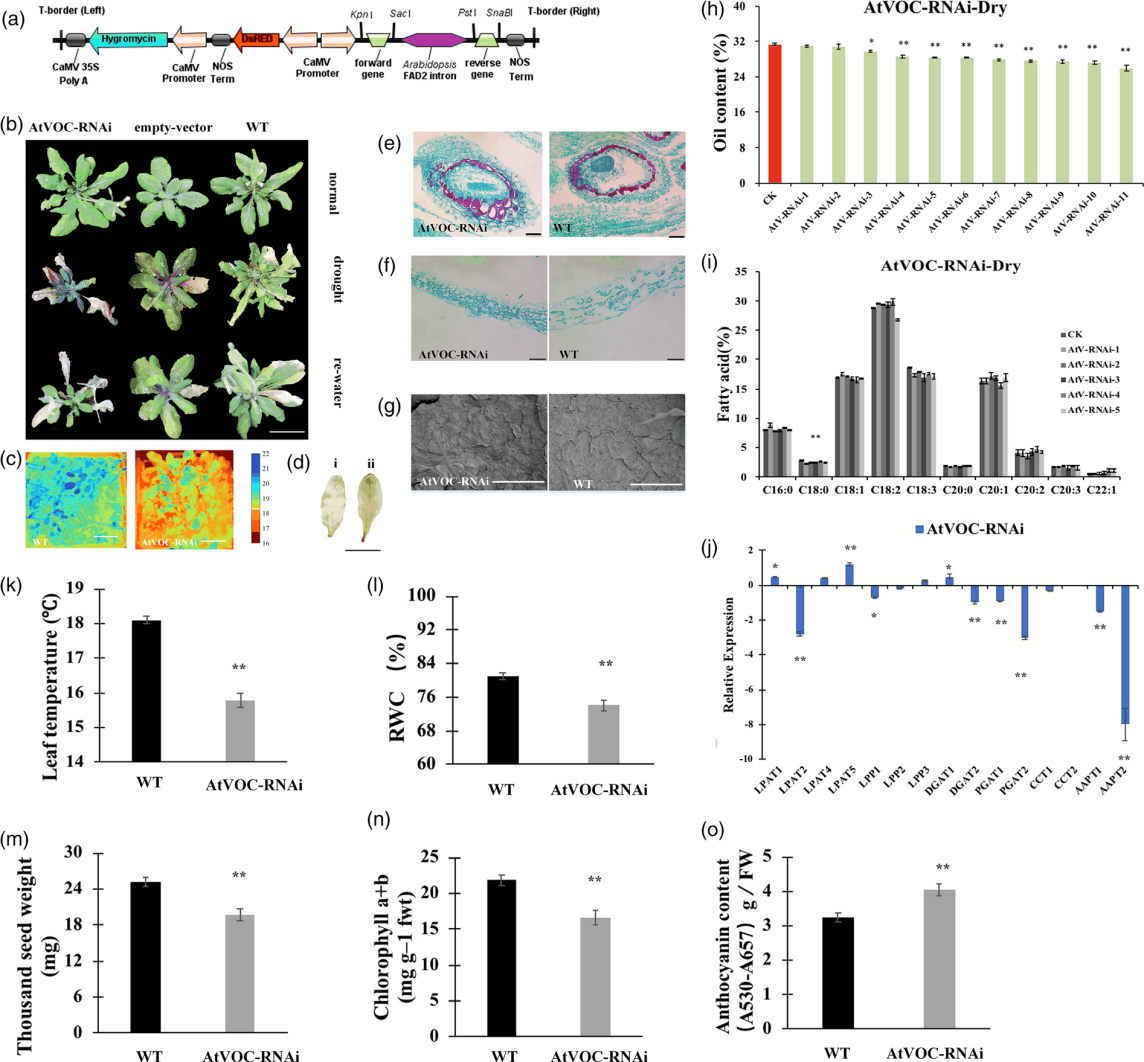
AtVOC‐RNAi lines have lower oil production and lower drought tolerance. (a): Schematic diagram of the RNA interference vector built in this study. (b): Phenotype of AtVOC‐RNAi and the empty vector and WT controls. (c): Thermal images of AtVOC‐RNAi and WT under drought conditions. (d): DAB staining of WT (i) and AtVOC‐RNAi (ii). Bar = 1 cm. The number below indicates the quantitative data of H_2_O_2_ content, and unit is nmol/g FW (e and f): Seed and leaf sections of WT and AtVOC‐RNAi under drought stress. Bar = 500 μm. (g): SEM observation of leaves of WT and AtVOC‐RNAi under drought conditions. Bar = 100 μm. (h): Oil content changes in the AtVOC‐RNAi lines. (i): FA composition of the AtVOC‐RNAi lines. (j): Relative expression levels of genes in the TAG synthetic pathway in AtVOC‐RNAi under drought stress. LPAT, lysophosphatidic acid acyltransferase; LPP, lipid phosphate phosphatase; DGAT, diacylglycerol acyltransferase; PDAT, phospholipid:diacylglycerol acyl transferase; CCT, choline‐phosphate; CTP, cytidylyltransferase; AAPT, aminoalcohol‐phosphotransferase. (k–o): Physiological indexes and TSWs of RNAi and WT under drought conditions. All the results are represented as the mean ± standard deviation (STD;* n* = 3). Statistically significant differences were determined using a two‐tailed paired Student's *t*‐test compared with WT plants under similar conditions, and the results are indicated by **P < 0.01 and *P < 0.05.

As the *LEA* and *VOC* genes were both associated with drought stress, the drought tolerance of the LEA3‐OE and VOC‐OE transgenic lines was first validated under drought treatment (Figure S1C). The T‐DNA insertion SALK *Arabidopsis* mutant (*atlea3*) (Figures 1a, S2) of the *AtLEA3* gene and the RNA interference line of *AtVOC* (AtVOC‐RNAi) (Figures 2a, S2) were used to further investigate the role of *AtLEA3* and *AtVOC* in drought tolerance and lipid accumulation. After being confirmed as homozygous by PCR and RT‐PCR, the *atlea3* materials were planted under normal conditions and subjected to drought treatment after flowering. The drought tolerances of the *atlea3* and AtVOC‐RNAi plants were analysed. The *atlea3* plants showed much weaker growth under drought treatment; they had lower relative water contents (RWCs) (Figure 1n), chlorophyll contents (Figure 1l), and leaf temperatures (Figure 1k) and higher anthocyanin contents (Figure 1o) and H_2_O_2_ accumulations (Figure 1c). The growth of the AtVOC‐RNAi plants was also affected by drought stress (Figure 2b–g). The AtVOC‐RNAi plants exhibited decreased drought tolerance with changes in the relevant physiological indexes compared with the wild‐type (WT) and empty vector controls (Figure 2k, l, n, o). A structural analysis of the seeds and leaves supported the conclusion of weaker drought tolerance (Figure 2e–g). The oil content of mature seeds in the mutant lines (24.5%) was 14.9% lower than that of WT (28.8%) under drought conditions (Figure 1i). Under normal conditions, the oil content of mature seeds in the mutant lines (27.5%) was 10.7% lower than that of WT (30.8%). C18:2 was also significantly affected by drought treatment in *atlea3* (Figure 1j). However, the oil content of the AtVOC‐RNAi lines was only slightly decreased in normal conditions and reduced by 5.3%–13.9% in the drought treatment (Figure 2h); a decrease in C18:0 was also observed (Figure 2i). Meanwhile, the thousand seed weights (TSWs) and seed sizes of *atlea3* and AtVOC‐RNAi were reduced by 10.6% (19.2 mg) and 8.7% (19.7 mg) compared with those of the control under drought conditions, respectively (Figures 1m, 2m). The expression of genes involved in lipid accumulation was down‐regulated in *atlea3* and AtVOC‐RNAi (Figures 1h, 2j). These results suggest that knockout of *AtLEA3* and knockdown of *AtVOC* could affect gene expression related to lipid accumulation.

The full‐length *AtLEA3* gene was cloned, and a complementation vector was constructed and then transformed into the *lea3* mutant (Figure 1). Compared with that of the mutant, the oil contents of the homozygous transgenic materials (AtLEA‐HBs) were rescued, with increases of 11.1%–25.3% and 12.9%–19.3% under drought and normal growth conditions, respectively (Figures 1b, S3), while the C18:1 contents of seeds in the AtLEA‐HBs were lower than those of *atlea3* (Figure 1j). In addition, the growth of the AtLEA3‐HBs under drought stress was recovered (Figure 1). These results suggested that loss or repression of these drought response genes could affect drought tolerance, lead to decreased oil content and reduce oil production.

Overall, these results indicated that overexpression of *LEA3* and *VOC* could enhance plant drought tolerance and maintain normal oil accumulation under drought conditions.

### Overexpression of the *BnLEA3*,* BnVOC*,* AtLEA3* and *AtVOC* genes could increase oil content and seed size in transgenic *Arabidopsis* under normal conditions

**Figure 3:**
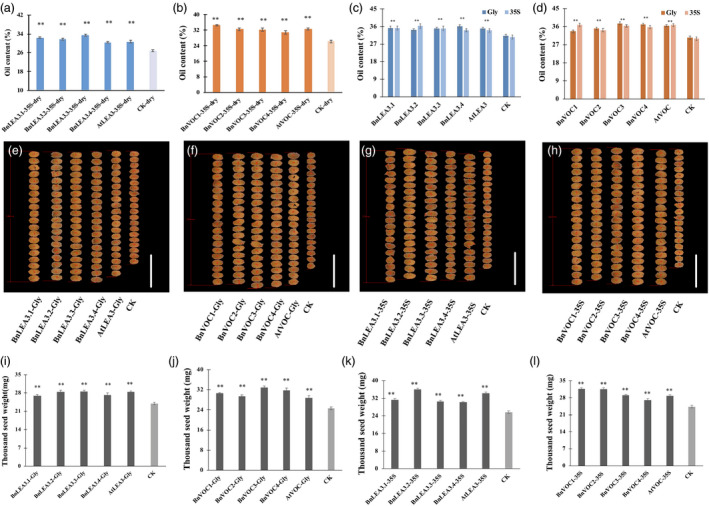
Overexpression of the *BnLEA3*,* BnVOC*,* AtLEA3* and *AtVOC* genes resulted in higher oil content, seed size and seed weight. (a and b): Oil contents of transgenic *Arabidopsis* seeds under drought conditions. (c and d): Oil contents of transgenic *Arabidopsis* seeds under normal conditions. (e–h): Seed sizes of LEA‐ and VOC‐OE lines (Gly and 35S) and CK. Bar = 1 mm. (i–l): TSWs of LEA‐ and VOC‐OE lines (Gly and 35S) and CK. The data represent the mean and standard deviation (STD) of three biological replicates (*n* = 3). Statistically significant differences were determined using a two‐tailed paired Student's *t*‐test compared with WT plants under similar conditions, and the results are indicated by **P < 0.01.

The oil content of transgenic lines in generation T3 was analysed, and the average oil contents of seeds in the BnLEA3.1‐Gly‐OE, BnLEA3.2‐Gly‐OE, BnLEA3.3‐Gly‐OE, BnLEA3.4‐Gly‐OE and AtLEA‐Gly‐OE transgenic lines were 34.2%–36.1%, 9.6%–15.7% higher than those of the control (CK, oil content: 31.2%; Figure 3). The maximum oil content in some transgenic lines was increased by 25% (Table S1). The seed oil contents of BnLEA3‐35S‐OE and AtLEA3‐35S‐OE were similar to those of LEA3‐Gly‐OE (Figure 3d), and the oil contents of BnLEA3.2‐35S‐OE and BnLEA3.3‐35S‐OE were as high as 34%–36.4%, an average increase of 18.3% (Table S1). The average oil contents of transgenic seeds from the BnVOC1‐Gly‐OE, BnVOC2‐Gly‐OE, BnVOC3‐Gly‐OE, BnVOC4‐Gly‐OE and AtVOC‐Gly‐OE lines increased from 10.8% to 23.9% (Figure 3d). The BnVOC‐35S‐OE and AtVOC‐35S‐OE lines also showed increased oil contents from 14% to 22.6% (Figure 3d). These results suggested that the *LEA3* and *VOC* genes take part in oil accumulation and help to increase seed oil content.

The sizes of the transgenic OE seeds were also measured. The seeds in the transgenic lines were larger than those of CK without morphological malformation (Figure 3e–h). Further analysis revealed that the TSW of LEA‐OE seeds was inconsistently increased by 4–9 mg. The LEA‐Gly‐OE lines showed a greater improvement in TSW than the LEA‐35S‐OE lines (Figure 3i, k). Compared with CK, the average TSWs of the BnLEA3‐Gly‐OE and AtLEA3‐Gly‐OE lines increased by 11.1%–19.1%, reaching 27–28.6 mg (Figure 3i). The TSWs of the BnVOC‐Gly‐OE lines ranged from 30.6 mg to 32.95 mg, with an average of 32 mg, which was higher than that of the BnVOC‐35S‐OE lines (TSWs: 27–31 mg; Figure 3j, l). These results indicated that overexpression of the *LEA3* and *VOC* genes could also improve seed size.

### Overexpression and RNA interference of the *BnLEA3* and *BnVOC* genes affect oil content, seed size and drought tolerance in *B. napus*

**Figure 4:**
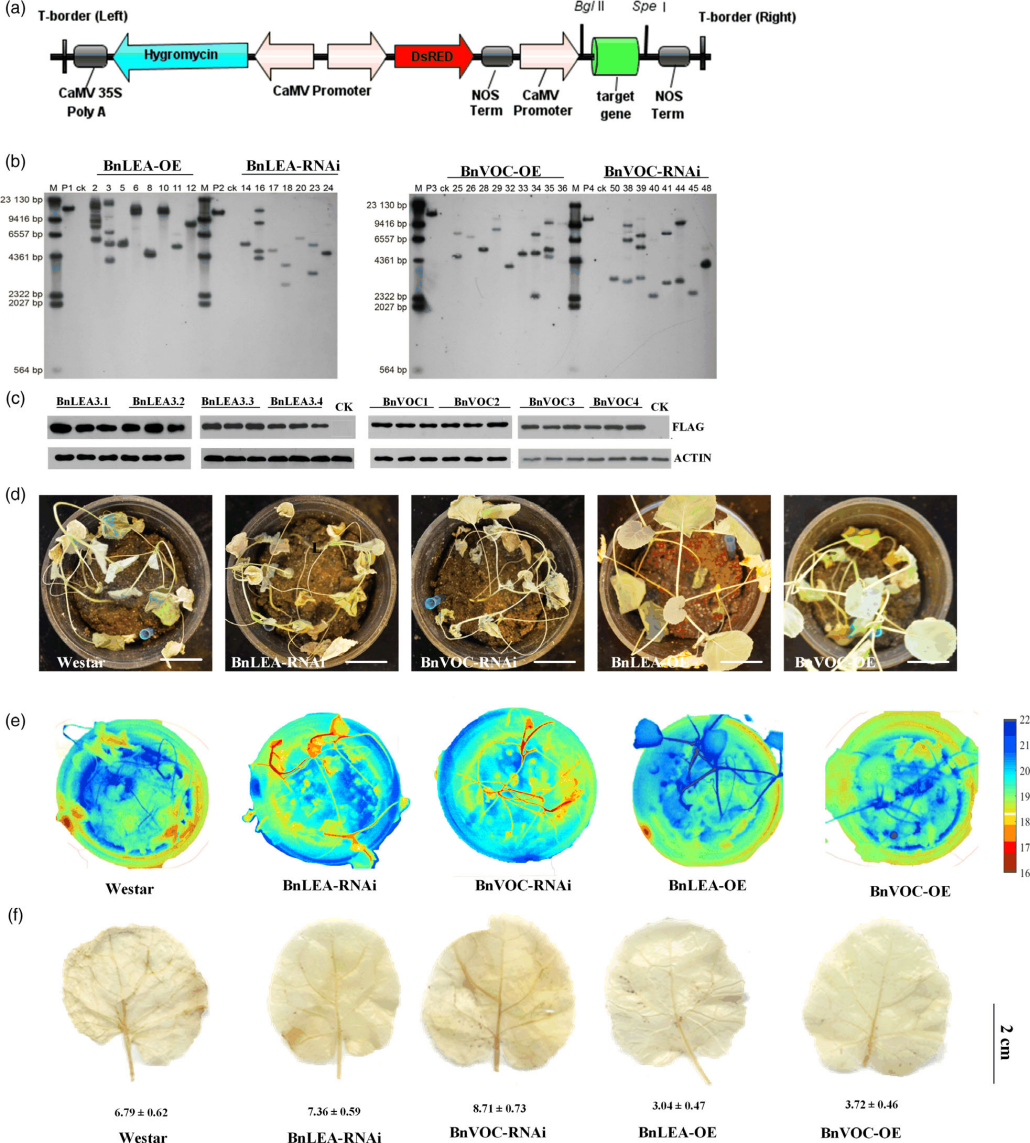
Transgenic *B. napus* showed improved drought tolerance. (a): Schematic diagram of the OE vector transformed into *B. napus* in this study. (b): Southern blotting was used to confirm the stable integration of the transgene in *B. napus*. (c): Western blotting was performed to check the expression of protein in WT (CK) and transgenic plants. (d): Phenotypes of transgenic plants and WT plants in the drought treatment. Bar = 5 cm. (e): Thermal images of transgenic and WT *B. napus* under drought conditions. (f): DAB staining of transgenic and WT *B. napus* under drought conditions. The number below indicates the quantitative data of H_2_O_2_ content, and unit is nmol/g FW. All the results are represented as the mean ± standard deviation (STD;* n* = 3).

To investigate the performance of the *BnLEA3* and *BnVOC* genes in *B. napus*, overexpression vectors containing four *BnLEA3*s (BnLEA‐OE) and four *BnVOC*s (BnVOC‐OE) and the corresponding RNA interference (BnLEA‐RNAi and BnVOC‐RNAi) vectors were constructed (Figures 4a, 2a) and then introduced into *B. napus*. The transgenic plants were confirmed by qPCR (Figure S4), Southern blotting and Western blotting (Figure 4b and c). The relative expression levels of the *LEA* and *VOC* genes in the OE and RNAi lines were 8‐ to 21‐fold higher and lower, respectively (Figure S4).

**Figure 5:**
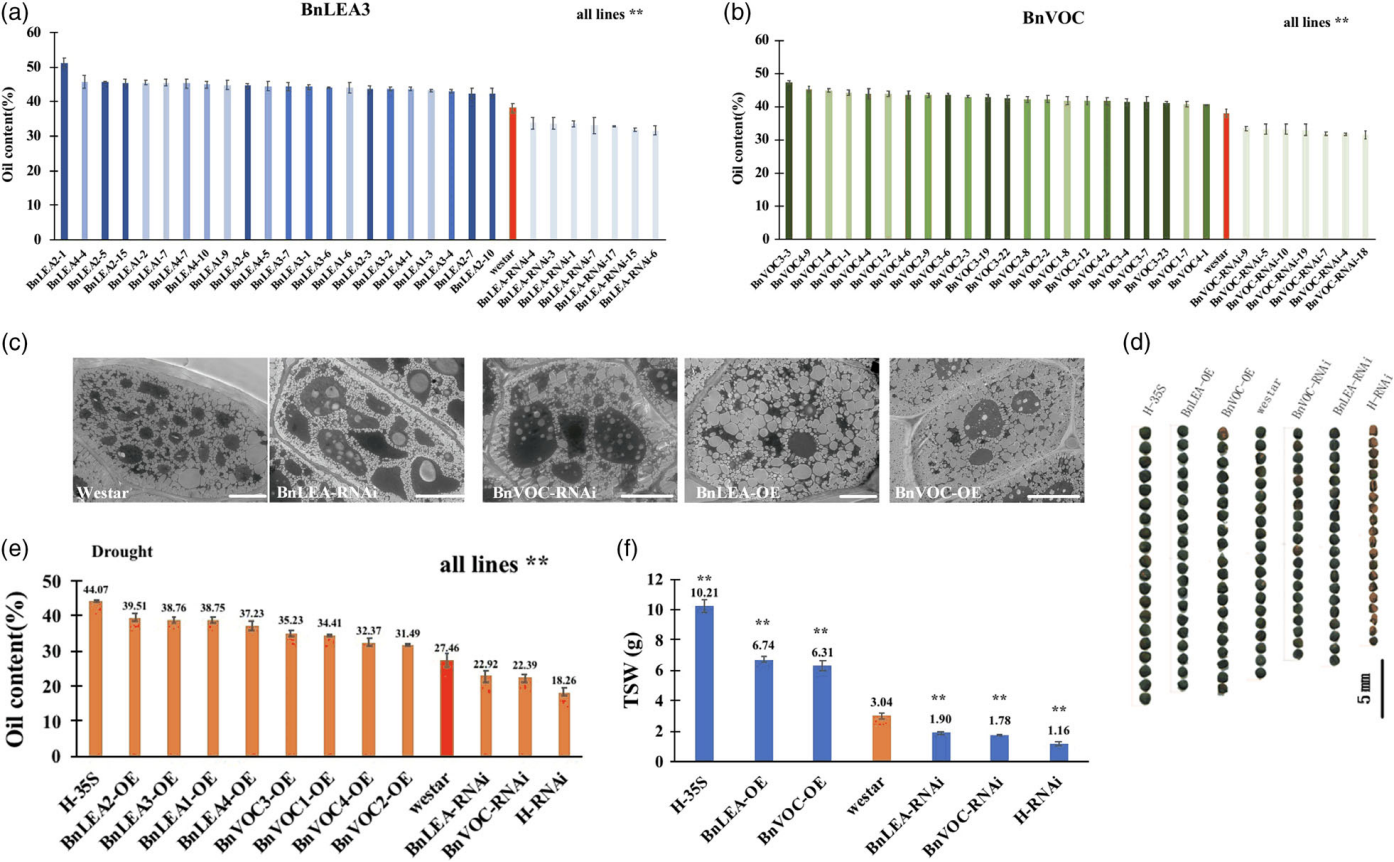
Transgenic *B. napus* showed changes in oil content and seed size. (a and b): Oil contents of transgenic *B. napus* lines. (c): TEM observation of transgenic and WT seeds. Bar = 10 μm. (d): Seed sizes of transgenic plants, hybrids (H‐35S: OE hybrids; H‐RNAi: RNAi hybrids) and WT. (e): Average oil contents of transgenic and hybrid plants under drought conditions. (f): TSWs of transgenic and hybrid plants. All the results are represented as the mean ± standard deviation (STD;* n* = 3). Statistically significant differences were determined using a two‐tailed paired Student's *t*‐test compared with WT plants under similar conditions, and the results are indicated by **P < 0.01.

Under drought stress, the BnLEA‐OE and BnVOC‐OE lines showed higher drought tolerance than the WT, BnLEA‐RNAi and BnVOC‐RNAi lines (Figure 4d). In addition, the RWCs and leaf temperatures greatly varied between the OE and RNAi lines and WT. The RWCs of BnLEA‐OE, BnVOC‐OE, BnLEA‐RNAi and BnVOC‐RNAi were 75.28%, 68.27%, 40.28% and 44.14%, respectively. The leaf temperatures of BnLEA‐OE and BnVOC‐OE were 20.5 and 19.9 °C, which were higher than those of WT (17.9 °C), BnLEA‐RNAi (16.8 °C) and BnVOC‐RNAi (16.1 °C) (Figure 4e). Further analysis showed that H_2_O_2_ accumulated more in the RNAi lines (Figure 5f). These results suggested that the *BnLEA* and *BnVOC* genes could also increase drought tolerance in *B. napus*.

Under normal condition, the oil content of WT was 37.6%, while the oil contents of the BnVOC‐OE and BnLEA‐OE lines were 43%–49.8% and 42%–48%, increases of 14.3%–32.9% and 11.7%–27.6%, respectively (Figure 5a and b). In contrast, the BnLEA‐RNAi and BnVOC‐RNAi lines had an average of 13.2%–15.9% decrease in oil content. In drought conditions, the oil contents of all lines were decreased, but those of the OE lines (above 30%) were higher than those of WT (27%). The oil content of BnLEA‐RNAi and BnVOC‐RNAi decreased more obviously, with an oil content of only 22%–23% (Figures 5e, S5). The changes in FA composition were mainly an increase in C18:1 and a decrease in C18:2 (Figure S6).

Further observation showed that the BnVOC‐OE and BnLEA‐OE lines had relatively large seed sizes with higher average TSWs (6.74 and 6.31 g, respectively) than WT. The seeds of the RNAi lines were much smaller than those of WT, with reduced average TSWs (only 1.9 and 1.78 g, respectively; Figure 5c and d). TEM observation showed that the seeds of both OE lines contained more oil bodies and less protein. In addition, the seeds of the RNAi lines showed fewer oil bodies, which were loosely arranged in the cells (Figure 5c). These results indicated that *BnLEA*s and *BnVOC*s play an important role in lipid accumulation and promote oil production.

### Transcriptome analyses of BnLEA‐OE, AtLEA‐OE and *atlea3* seeds reveal distinct patterns of gene activity and regulatory networks affecting oil metabolism

**Figure 6:**
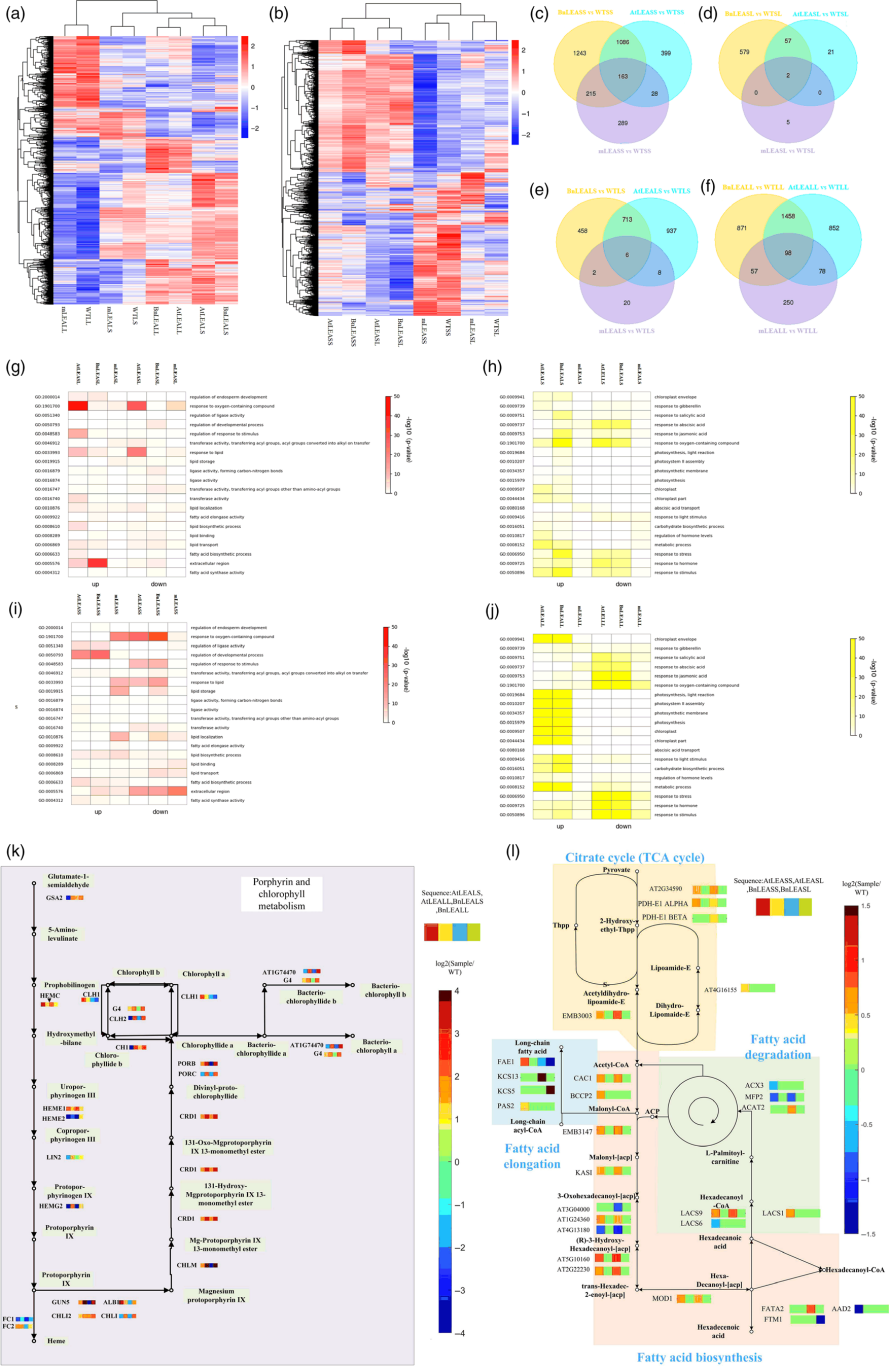
RNA‐seq analysis of BnLEA‐OE, AtLEA‐OE,* atlea3* and WT under short‐ and long‐term drought stress. (a and b): Cluster heatmaps of DEGs in the leaves (a) and seeds (b) of plants differentially expressing LEA under drought conditions. (c–f): Venn diagrams showing the numbers of DEGs in different genotypes of *Arabidopsis* (*P* ≦ 0.05, false discovery rate < 0.05). (g–j): Significantly enriched GO terms (coloured in red and yellow) of the DEGs in the seeds of differential LEA expression lines in the long‐term (g) and short‐term (i) drought treatments and the corresponding leaves in the long‐term (j) and short‐term (h) drought treatments. (k and l): Major enriched KEGG pathways and the expression of the related genes in the leaves (k) and seeds (l) of plants differentially expressing LEA.

RNA‐seq analysis was performed to explore the regulatory mechanisms active in seeds under drought stress. To investigate the function of the *LEA* and *VOC* genes in drought response and drought tolerance, two types of drought treatment were performed: short‐term drought treatment to observe the response when plants initially encountered drought and long‐term drought treatment to observe tolerance when plants were under prolonged drought stress (Table 1). In total, 8 groups of lines were subjected to transcriptome analysis, including BnLEASL, AtLEASL, mLEASL, BnLEASS, AtLEASS and mLEASS. WTSL and WTSS were used as controls (the first letter ‘S’ indicates a seed sample, and for the second letter, ‘L’ and ‘S’ represent the long‐term and short‐term drought treatments, respectively; the initial ‘m’ indicates mutant). The gene sets that were activated in these genotypes in response to drought were identified (Figure 6b). In the short‐term drought treatment, compared with WTSS, 1676, 2707 and 695 differentially expressed genes (DEGs) were detected in AtLEASS, BnLEASS and mLEASS (Figure 6c and d). Further analysis revealed that 163 DEGs were the same in all these lines. In the long‐term drought treatment, only 80, 638 and 7 DEGs were found in AtLEASL, BnLEASL and mLEASL, respectively. These results suggested that there were large, coordinated shifts in gene expression at the early stage of drought stress in the OE lines and mutants.

**Table 1:** RNA‐seq analysis codes for the drought treatments combining the genotype and sample tissues

Code	Genotype	Drought treatment	Sample tissue
AtLEALL	Over expression of AtLEA	Long‐term	Leaves
BnLEALL	Over expression of BnLEA	Long‐term	Leaves
AtVOCLL	Over expression of AtVOC	Long‐term	Leaves
BnVOCLL	Over expression of BnVOC	Long‐term	Leaves
mLEALL	Mutant of AtLEA	Long‐term	Leaves
AtVOCRNAiLL	RNAi of AtVOC	Long‐term	Leaves
WTLL	Wild type	Long‐term	Leaves
AtLEALS	Over expression of AtLEA	Short‐term	Leaves
BnLEALS	Over expression of BnLEA	Short‐term	Leaves
AtVOCLS	Over expression of AtVOC	Short‐term	Leaves
BnVOCLS	Over expression of BnVOC	Short‐term	Leaves
mLEALS	Mutant of AtLEA	Short‐term	leaves
AtVOCRNAiLS	RNAi of AtVOC	Short‐term	Leaves
WTLS	Wild type	Short‐term	Leaves
AtLEASL	Over expression of AtLEA	Long‐term	Silique
BnLEASL	Over expression of BnLEA	Long‐term	Silique
AtVOCSL	Over expression of AtVOC	Long‐term	Silique
BnVOCSL	Over expression of BnVOC	Long‐term	Silique
mLEASL	Mutant of AtLEA	Long‐term	Silique
AtVOCRNAiSL	RNAi of AtVOC	Long‐term	Silique
WTSL	Wild type	Long‐term	Silique
AtLEASS	Over expression of AtLEA	Short‐term	Silique
BnLEASS	Over expression of BnLEA	Short‐term	Silique
AtVOCSS	Over expression of AtVOC	Short‐term	Silique
BnVOCSS	Over expression of BnVOC	Short‐term	Silique
mLEASS	Mutant of AtLEA	Short‐term	Silique
AtVOCRNAiSS	RNAi of AtVOC	Short‐term	Silique
WTSS	Wild type	Short‐term	Silique

To examine the biological processes encoded within differentially expressed gene sets, the Gene Ontology (GO) enrichment function of SeqEnrich was used (Figure 6g, i). DNA replication and cell proliferation were significantly up‐regulated in both OE lines under short‐term drought; many genes showed increased expression, and cell growth processes were the most strongly up‐regulated. In *atlea3*, genes related to response to oxygen‐containing compound and abiotic stimuli were up‐regulated in the short‐term drought treatment (Figure 6g, i). These results suggested that other abiotic stress response pathways were still able to respond to the threat of drought without the *AtLEA3* gene. In the long‐term drought treatment, genes related to extracellular region, regulation of endosperm development and lipid transport were mainly up‐regulated in the OE lines, and response to oxygen‐containing compound and stimulus genes were down‐regulated in the mutant lines. These results suggest that, because overexpression of *LEA3* genes can increase the expression of genes involved in lipid‐related processes, a loss of *LEA*3 might lead to desiccation tolerance in long‐term drought. To further identify potential regulatory networks, Kyoto Encyclopedia of Genes and Genomes (KEGG) enrichment analysis was performed (Figure 6l). In the LEA‐OE lines, FA biosynthesis was enriched and FA degradation was depleted. This result indicated that LEA overexpression could contribute to oil accumulation.

### Transcriptome changes in LEA‐OE and *atlea3* identified that photosynthetic carbon fixation confers drought tolerance in leaves

To study the gene expression changes of leaves under drought conditions, the leaves of BnLEA‐OE, AtLEA‐OE, *atlea3* and WT under drought treatment were subjected to RNA‐seq analysis (BnLEALL, AtLEALL mLEALL, WTLL, BnLEALS, AtLEALS mLEALS, WTLS: the first letter ‘L’ indicates a leaf sample, and for the second letter, ‘L’ and ‘S’ indicate the long‐term and short‐term drought treatments, respectively; Figure 6a). Compared to WT, 98 DEGs were commonly found in BnLEALL, AtLEALL and mLEALL (Figure 6f), but only 6 DEGs were common among these three genotypes in the short‐term drought treatment (AUXIN ASSOCIATED GENE 2, leucine‐rich repeat family protein, transmembrane amino acid transporter family protein, transposable_element_gene, AT5G38005 and RIBOSOMAL RNA26S; Figure 6e). These results demonstrated that more genes were differentially expressed in leaves in the long‐term drought treatment than in the short‐term drought treatment. The GO analysis showed that genes associated with response to oxygen‐containing compound, stress and stimulus were affected in OE lines in the early stage. Chloroplast part and plastid part were enriched in OE lines in the long‐term drought treatment. However, cutin biosynthetic process and structural constituent of the cell wall were reduced in the mutant in the short‐term drought treatment. Response to oxygen‐containing compound was significantly enriched in the mutant in the long‐term drought treatment (Figure 6h, j). These results indicated that the LEA‐OE lines could quickly enrich biological processes involved in stress response in the early stages of drought and keep chloroplasts and other parts of the cell stable under long‐term drought. In *atlea3*, this genetic response to stress was delayed, and the plants lacked the ability to stabilize their organelles. KEGG analyses showed that carbon fixation in photosynthetic organisms and chlorophyll metabolism were up‐regulated in the OE lines (Figure 6k), which indicated that *LEA* could confer greater photosynthetic capacity under drought stress.

### Transcriptome analyses of BnVOC‐OE, AtVOC‐OE and AtVOC‐RNAi lines reveal *VOC* function in FA degradation in seeds

**Figure 7:**
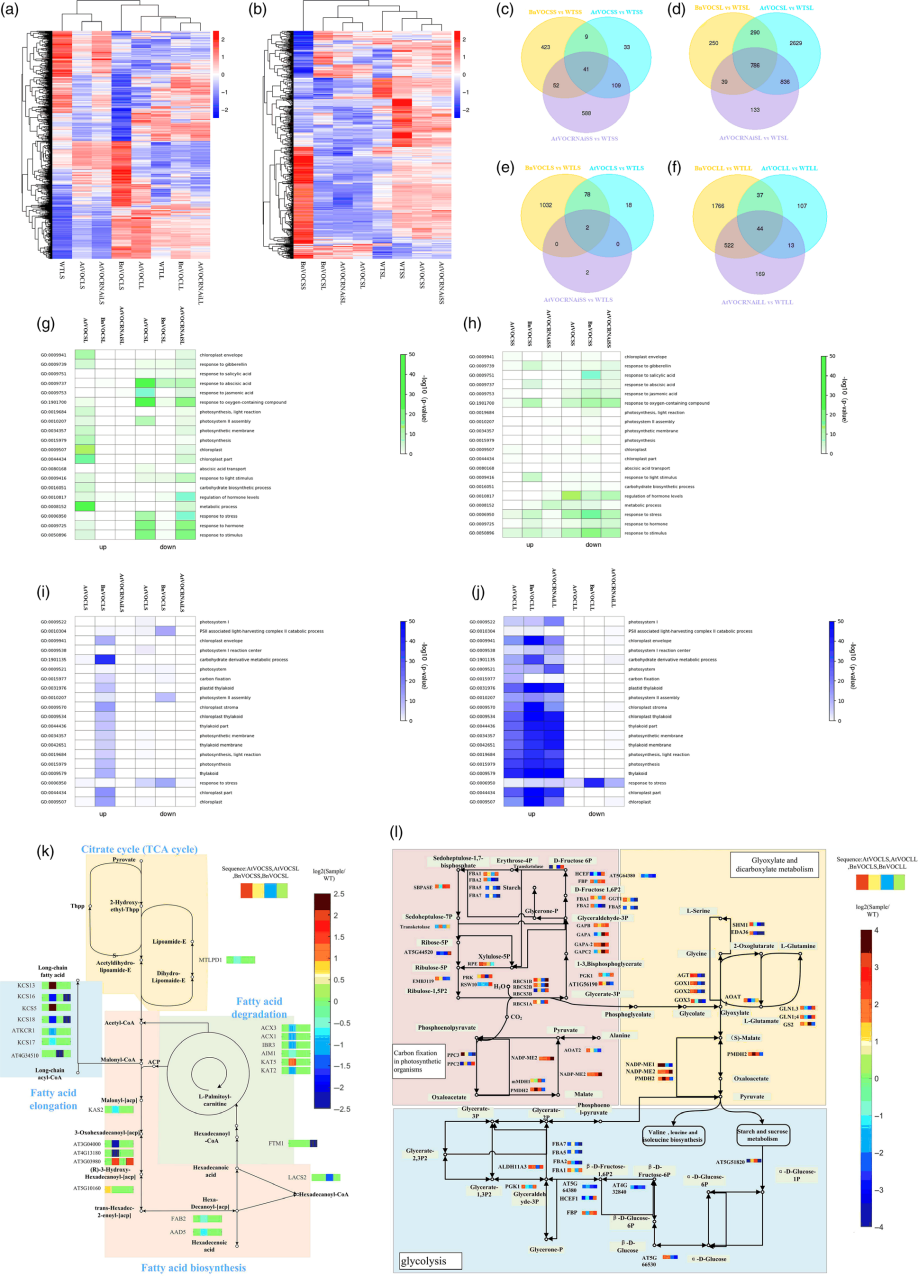
Transcriptome analysis of plants differentially expressing VOC and WT plants under short‐ and long‐term drought stress. (a and b): Cluster heatmaps of DEGs in the leaves (a) and seeds (b) of plants differentially expressing VOC under drought conditions. (c–f): Venn diagrams showing the numbers of DEGs in different genotypes of *Arabidopsis* (*P *≦ 0.05, false discovery rate < 0.05). (g–j): Significantly enriched GO terms (coloured in red and yellow) of the DEGs in the seeds of differential VOC expression lines in the long‐term (g) and short‐term (h) drought treatments and the corresponding leaves in the long‐term (j) and short‐term (i) drought treatments. (k and l): Major enriched KEGG pathways and the expression of the related genes in the seeds (k) and leaves (l) of plants differentially expressing VOC.

To identify the potential mechanism of VOC action in drought treatment, RNA‐seq of seeds in the OE and RNAi transgenic lines was conducted in two drought treatments (BnVOCSL, AtVOCSL, AtVOCRNAiSL, BnVOCSS, AtVOCSS, AtVOCRNAiSS: the first letter ‘S’ indicates a seed sample; for the second letter, ‘L’ and ‘S’ represent the long‐term and short‐term drought treatments, respectively; Figure 7b). In the short‐term drought treatment, 41 and 786 common DEGs in these three genotypes in the early stage and late stage were identified compared with WT, respectively (Figure 7c and d). These results suggest that VOC genes tend to affect late seed development under drought conditions. The GO analysis of BnVOCSS showed enrichment in response to stress and oxygen‐containing compound. In AtVOCSS, auxin metabolic process was enriched. In AtVOCRNAiSS, DNA replication was significantly enriched. In the long‐term drought treatment, genes involved in seed maturation, response to stimulus, lipid localization, seed oil body biogenesis and lipid storage were active in the BnVOC‐OE and AtVOC‐OE lines. However, response to abiotic stimulus, oxygen‐containing compound and water were down‐regulated in AtVOCRNAiSL (Figure 7g–h).

Kyoto Encyclopedia of Genes and Genomes analysis revealed that the VOC genes take an active part in FA degradation and FA elongation and that many genes involved in FA degradation were down‐regulated in the VOC‐OE lines. The expression levels of lipid metabolism genes in the AtVOC‐RNAi lines showed opposite trends to those in the AtVOC‐OE lines. These results demonstrated that VOC could function in lipid metabolism by reducing FA degradation (Figure 7k).

### Glyoxylate metabolism is significantly affected in the leaves of VOC transgenic plants

To further investigate the role of the VOC gene in response to drought, RNA‐seq analysis was performed with the leaves of VOC transgenic plants in short‐ and long‐term drought treatment (BnVOCLL, AtVOCLL, AtVOCRNAiLL, BnVOCLS, AtVOCLS, AtVOCRNAiLS: the first letter ‘L’ after the gene/construct name indicates a leaf sample, and for the second letter, ‘L’ and ‘S’ indicate the long‐term and short‐term drought treatments, respectively; Figure 7a). Compared with WTLL, 44 common DEGs were found in BnVOCLL, AtVOCLL and AtVOCRNAiLL, but only 2 common DEGs were found in BnVOCLS, AtVOCLS and AtVOCRNAiLS (AT1G15380: GLYI4; AT3G59930: a defensin‐like (DEFL) family protein; Figure 7e and f). These results indicated that genes affected by VOC were mainly expressed in the long‐term drought treatment. The GO categories enriched in BnVOCLS and AtVOCLS were mainly ribonucleotide biosynthetic process and response to ROS. In AtVOCRNAiLS, response to hormone was enriched. In the long‐term drought treatment, the BnVOCLL lines showed enrichment of response to oxygen‐containing compound and chloroplast part. The GO categories enriched in AtVOCLL and AtVOCRNAiLL were mainly photosynthesis and light reaction (Figure 7i and j). These results suggested that BnVOC and AtVOC differed under both short‐ and long‐term drought conditions but consistently affected light reactions and hormones. KEGG pathway analysis showed that glyoxylate and dicarboxylate metabolism and plant hormone signal transduction were enriched in OE transgenic leaves (Figure 7l). The BnVOC‐OE and AtVOC‐OE lines contained more up‐regulated genes in the glyoxylate pathway than the AtVOC‐RNAi lines. These results indicated that glyoxylate metabolism and oxygen‐related metabolic process are affected by *VOC* genes.

### Hybrids of LEA and VOC transgenic lines had altered oil content, seed size and drought tolerance in *B. napus* and *Arabidopsis*

As mentioned above, both LEA and VOC could improve oil content and stress tolerance under drought conditions, but these genes had different regulatory mechanisms. To further investigate whether they could work together to strengthen drought tolerance or oil production under drought conditions, we hybridized BnLEA‐OE with BnVOC‐OE (H‐35S) and BnLEA‐RNAi with BnVOC‐RNAi (H‐RNAi), and also cross BnLEA‐OE with BnVOC‐RNAi in *B. napus*. We also crossed the transgenic *Arabidopsis* BnLEA‐35S line with BnVOC‐35S (H‐Bn‐OE), AtLEA‐35S with AtVOC‐35S (H‐At‐OE) and *atlea3* with AtVOC‐RNAi (H‐mi) in *Arabidopsis*.

**Figure 8:**
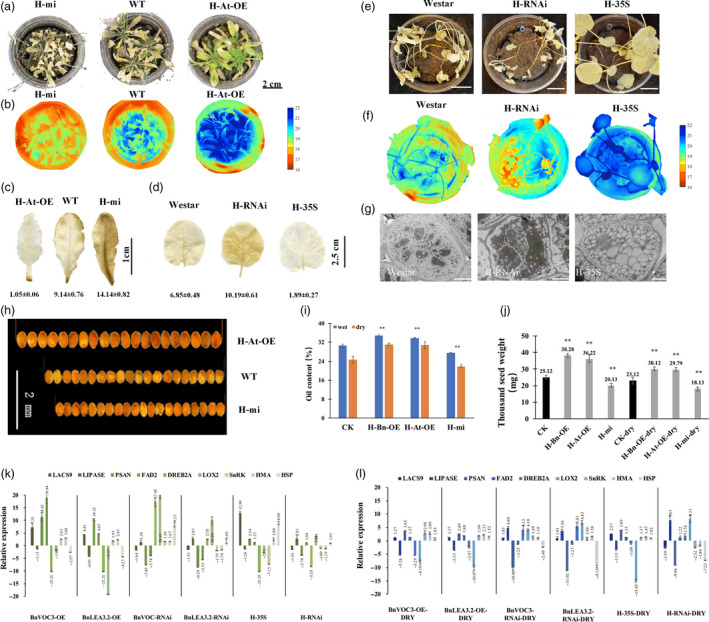
Hybrids of LEA and VOC in *B. napus* and *Arabidopsis*. (a): Performance of *Arabidopsis* hybrids in the drought treatment. (b): Thermal images of *Arabidopsis* hybrids in the drought treatment. (c and d): DAB staining of *Arabidopsis* (c) and *B. napus* (d) hybrids in the drought treatment. The number below indicates the quantitative data of H_2_O_2_ content, and unit is nmol/g FW. (E): Performance of *B. napus* hybrids in the drought treatment. Bar = 5 cm. (f): Thermal images of *B. napus* hybrids in the drought treatment. (g): SEM observations of *B. napus* hybrids in the drought treatment. Bar = 5 μm. (h): Seed sizes of *Arabidopsis* hybrids. (i): Oil contents of *Arabidopsis* hybrids. (j): TSWs of *Arabidopsis* hybrids. (k and l): Relative expression levels of key genes in transgenic and hybrid *B. napus* under normal conditions (k) and drought conditions (l). All the results are represented as the mean ± standard deviation (STD;* n* = 3). Statistically significant differences were determined using a two‐tailed paired Student's *t*‐test compared with WT plants under similar conditions, and the results are indicated by **P < 0.01.

In *B. napus*, the hybrids of OE and RNAi showed nearly 10% changes in oil content (highest in H‐35S: 51.15%; lowest in H‐RNAi: 27.23%) under normal conditions (Figure 5). After drought treatment, the growth of the hybrids was affected, and H‐35S showed better drought tolerance than the H‐RNAi lines (Figure 8a, d, e). Under drought conditions, the oil content of H‐RNAi was only 18.26%; in contrast, H‐35S had an oil content of 44.07%. The TSW of H‐35S could be improved to 10.21 g. The average TSW of the H‐RNAi lines was greatly diminished to only 1.16 g under drought conditions (Figure 5f). Scanning electron microscope (SEM) observation found that the oil bodies of the hybrids showed corresponding variations: the number of oil bodies in H‐35S was increased, and the oil bodies were closely arranged. In contrast, the protein bodies were fewer than in WT. In contrast, the seeds of the H‐RNAi lines contained fewer oil bodies and more protein bodies than those of WT (Figure 8g).

In *Arabidopsis*, the H‐Bn‐OE lines had a higher oil content than H‐At‐OE and H‐mi under both normal and drought conditions. The oil content of H‐At‐OE was 31.8%, while the oil content of H‐mi was only 21.9% under drought conditions (Figure 8i). The FA composition was also altered under drought conditions (Figure S7). The TSW of H‐Bn‐OE was increased by 28.8%, and the H‐mi was decreased by 27.52% under drought conditions (Figure 8j). Further analysis showed that the drought tolerance of H‐Bn‐OE and H‐At‐OE was much better than that of H‐mi (Figure 8a–c). The seeds of the OE hybrids and RNAi/mutant hybrids had larger and smaller seed sizes, respectively, in both *B. napus* and *Arabidopsis* than those of CK (Figure 8h). The expression of key genes in the regulatory pathways of FA metabolism, oxidation and photosynthesis in the hybrids and transgenic lines showed that the hybrids had higher expression levels of positive‐effect genes and lower expression levels of negative‐effect genes in drought response and lipid accumulation (Figure 8k and l). The hybrid combination of LEA‐OE and VOC‐RNAi shows the similar level with WT. These results demonstrated that *LEA* and *VOC* have different mechanisms in response to drought, and their effects could be reinforced or weakened in hybrids.

### Transgenic and hybrid OE and RNAi *B. napus* lines maintain altered photosynthetic machinery under drought stress

**Figure 9:**
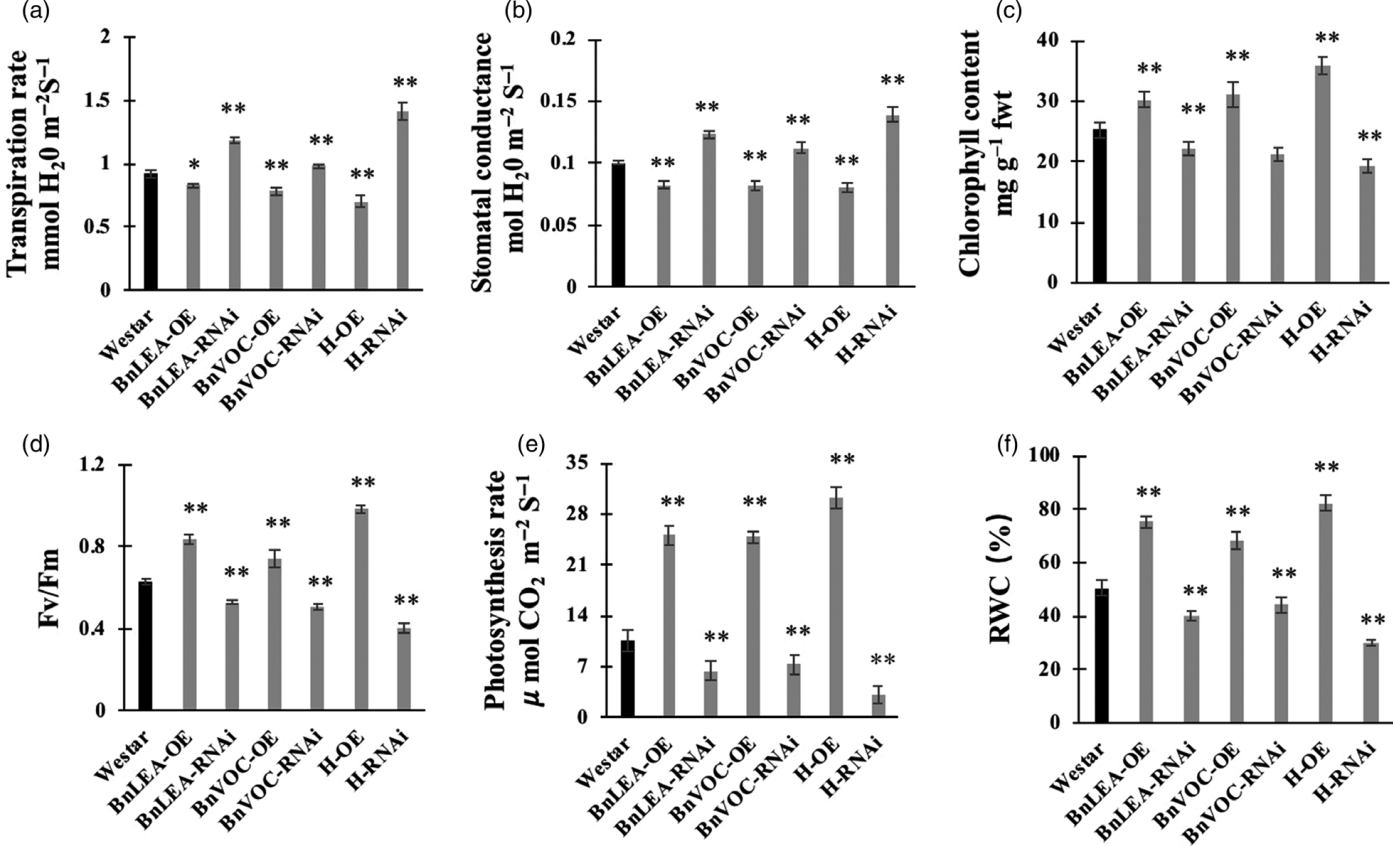
Photosynthetic efficiency of transgenic *B. napus* under drought stress. (a–f): Comparison of various photosynthetic and physiological parameters of WT and transgenic oilseed rape under drought conditions. (a) Transpiration rate, (b) stomatal conductance, (c) chlorophyll content, (d) *Fv*/*Fm*, (e) photosynthetic rate and (f) RWC were measured. All the results are represented as the mean ± standard deviation (STD;* n* = 3). Statistically significant differences were determined using a two‐tailed paired Student's *t*‐test compared with WT plants under similar conditions, and the results are indicated by **P < 0.01 and *P < 0.05.

The transcriptome results showed that the *LEA* and *VOC* genes could affect photosynthesis and FA metabolism in the transgenic lines and hybrids of LEA‐OE and VOC‐OE *B. napu*s lines. In addition, the BnLEA‐OE and BnVOC‐OE lines and the OE hybrids had better drought tolerance and higher oil contents than WT. Various determinants of photosynthesis were measured in the drought treatment (Figure 9). The BnLEA‐OE and BnVOC‐OE transgenic lines and the 35S hybrid lines all showed higher chlorophyll content, higher photosynthetic rate and higher RWC than WT under drought conditions. Other parameters in these transgenic and hybrid plants further supported their ability to maintain better photosynthetic capacity under drought stress. On the other hand, the BnLEA‐RNAi, BnVOC‐RNAi, and RNAi hybrids all had lower chlorophyll contents and photosynthetic rates. These results indicated that the LEA and VOC genes could help to improve photosynthetic efficiency under drought conditions.

Combining the regulatory pathway information gathered from the many transcriptome results and experiments shown above, we concluded that the LEA genes could enhance oil accumulation mainly via improved photosynthetic efficiency. As shown in Figure 11, we can deduce that the LEA‐OE lines contained more chlorophyll and had a higher photosynthetic rate, ensuring sucrose synthesis under drought stress, which provided energy and an upstream substrate for FA metabolism. Second, *LEA* affected many relative proteins during FA metabolism in the plastid and endoplasmic reticulum, such as LACS9, acyl carrier proteins to enhance FA biosynthesis, and LOX and LIPASE to decrease lipid oxidation and degradation. Third, the LEA‐OE lines had altered levels of heavy metal‐associated proteins, SnRK and plant lipid transfer proteins (LTP), which could increase plant cell integrity by reducing metal toxicity, reducing stomatal aperture and enhancing membrane stability.

### Reduced ROS level, oxidation system activity and enhanced MG tolerance conferred drought tolerance on BnVOC transgenic and hybrid *B. napus*

The identification of enriched redox‐related GO terms and DEGs prompted us to study additional differences in the redox system between the VOC lines and WT under drought stress. *VOC* genes play an important role in MG detoxification reactions, and MG tolerance was confirmed by experiments. The results of prokaryotic expression showed that VOC proteins could confer MG tolerance in *E. coli* (Figure S8). Under drought stress, the contents of H_2_O_2_ in the *B. napus* BnVOC‐OE and BnVOC‐RNAi lines were reduced and increased, respectively, compared with those of WT. MG, a cytotoxic by‐product of the glycolytic pathway, also had a lower content in the BnVOC‐OE lines.

**Figure 10:**
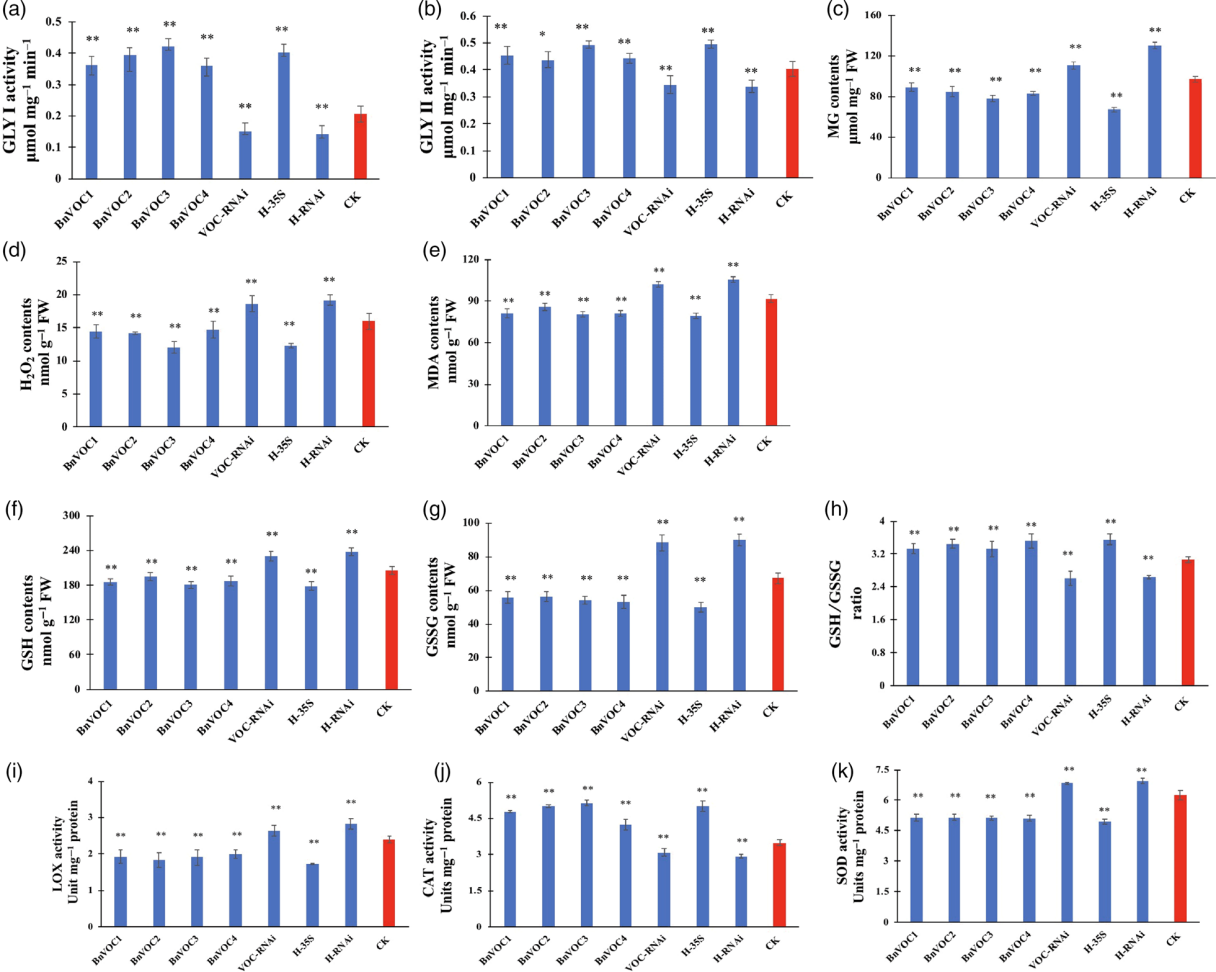
Activity of the oxidation system and glyoxalase system of *BnVOC* transgenic *B. napus* under drought stress. (a–k): GLY I activity (a), GLY II activity (b), MG content (c), H_2_O_2_ content (d), MDA content (e), GSH content (f), GSSG content (g), GSH/GSSG ratio (h), LOX activity (i), CAT activity (j) and SOD activity (k) in transgenic and hybrid *B. napus* under drought stress. All the results are represented as the mean ± standard deviation (STD;* n* = 3). Statistically significant differences were determined using a two‐tailed paired Student's *t*‐test compared with WT plants under similar conditions, and the results are indicated by **P < 0.01 and *P < 0.05.

**Figure 11:**
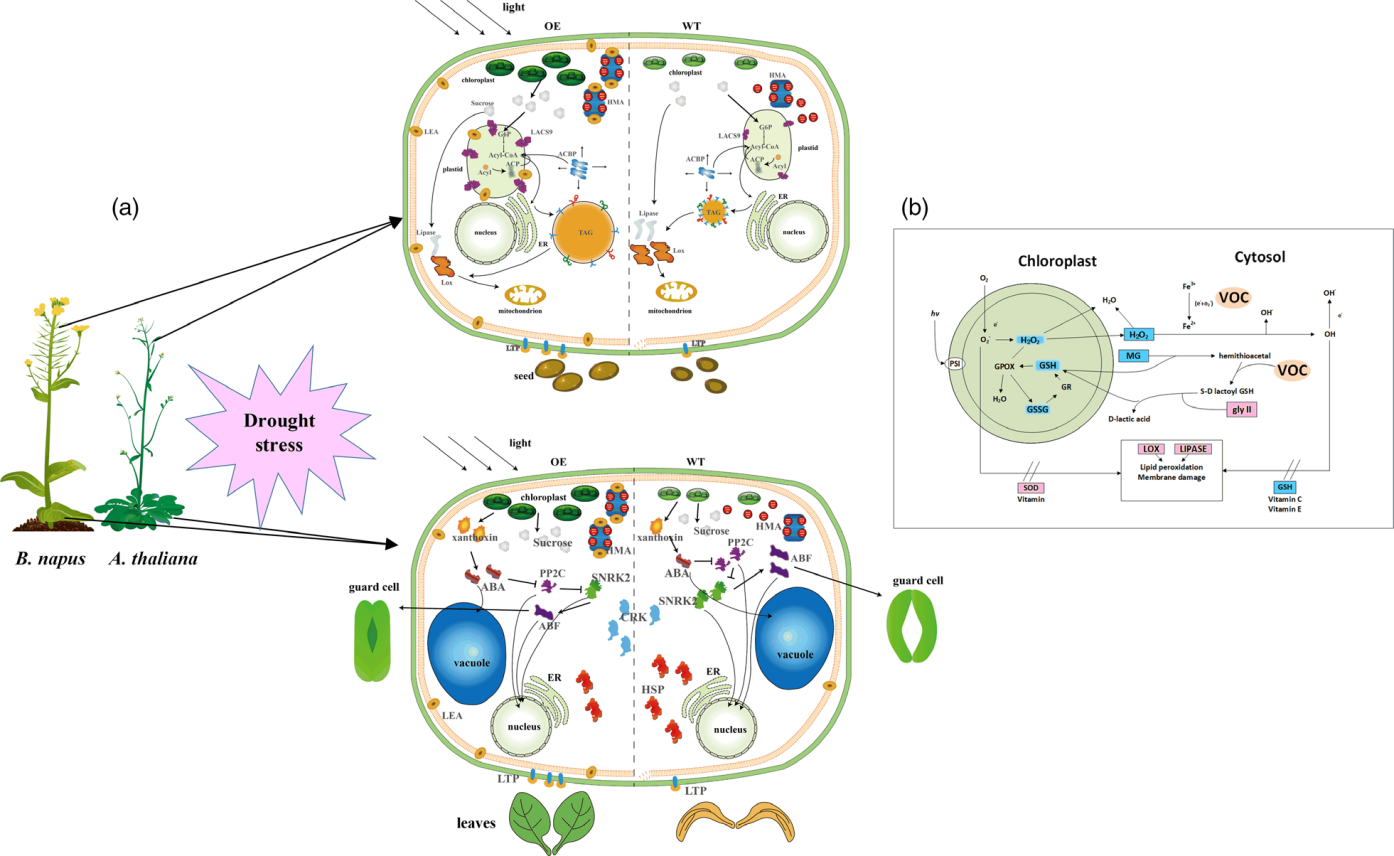
Model of LEA and VOC functional regulation in plants. (a): Schematic representation of the influence of LEA genes on FA metabolic and catabolic biosynthetic pathways under drought stress. Photosynthetic efficiency, membrane stability and lipid synthesis and transportation are improved under drought conditions, resulting in higher seed oil contents in *B. napus* and *Arabidopsis*. The plant hormone signalling pathways lead to stomatal closure, improved membrane stability and reduced metal ion toxicity, conferring drought tolerance in OE leaves. HMA, heavy metal‐associated domain protein; G6P, glucose‐6‐phosphate; ACBP, Acyl‐CoA binding proteins; ER, endoplasmic reticulum; ACP, acyl carrier protein; LACS9, long‐chain acyl‐CoA synthetase 9; HSP, heat shock factor protein. (b): The effect of VOC genes on the ROS system reduces H_2_O_2_ and MG contents, which improves oil content and drought tolerance by reducing lipid peroxidation and membrane damage. ABF, ABRE binding factors; ABA, abscisic acid; PP2C, protein phosphatase 2C; SNRK2, SNF‐related kinase 2; CRK, cysteine‐rich receptor‐like kinase; hv, irradiation with light; PS I, photosystem I; GPOX, glutathione peroxidase; GR, glutathione reductase.

In the glyoxalase system, GLY I and glyoxalase II (GLY II) catalyse the MG detoxification reactions. The main subfamily of VOCs is GLY I. It was found that the activity of GLY I significantly increased, and the activity of GLY II also improved in the BnVOC‐OE lines (Figure 10). In addition, the BnVOC‐OE lines had a lower content of malondialdehyde (MDA, a product of lipid peroxidation). GSH and oxidized glutathione (GSSG) contents were also changed under drought conditions. Furthermore, lipoxygenase (LOX) and superoxide dismutase (SOD) activities were increased in the BnVOC‐RNAi lines and decreased in the BnVOC‐OE lines. Catalase (CAT) activity was inversely correlated with those of SOD and LOX. The results for the H‐35S and H‐RNAi hybrids were consistent with those for the OE and RNAi lines (Figure 11). Based on the results above and the transcriptome results, the molecular pathway of VOC in drought conditions was deduced (Figure 11). First, VOC proteins could definitely reduce MG toxicity through glyoxylate metabolism with GSH. VOC proteins also contributed to lower levels of ROS synthesis (H_2_O_2_). Finally, lipid peroxidation and membrane damage were reduced.

## Discussion

Drought tolerance was the first obvious change in the *LEA* and *VOC* differential expression lines. Stomata regulate the flow of gases and transpiration by limiting water loss in different environments (Hetherington and Woodward, 2003). In this study, the stomata in the LEA and VOC‐OE lines tended to be closed under drought conditions, as observed in other plants (He *et al*., 2018; Prasch *et al*., 2015; Ullah *et al*., 2017). To study the lipid changes associated with drought stress, the oil content of transgenic plants under drought conditions was also analysed. The oil content of WT was only 25.3%–26.6% (Figure 3a and b), a reduction of 14.1%–18.3% compared with the control, under drought stress. However, the oil contents of the LEA3‐OE and VOC‐OE lines were 30.9%–34.6% (Figure 3a) and 30.7%–33.5% (Figure 3b), respectively. These results suggested that *LEA3* and *VOC* could stabilize oil accumulation under drought stress. The morphology of the OE and WT plants under drought stress was observed by paraffin sectioning and SEM analysis (Figure S9). The OE seeds developed normally, while the WT seeds became wizened under drought stress. Meanwhile, the leaves of WT were more wrinkled than the OE leaves, and stomatal closure was more pronounced in the OE leaves. These results indicated that *LEA3* and *VOC* can maintain normal seed and leaf development under drought stress. The enhanced drought tolerance of the LEA3‐35S‐OE and VOC‐35S‐OE lines was also reflected by physiological index analysis. The chlorophyll contents and RWCs of the LEA3‐35S‐OE and VOC‐35S‐OE lines were significantly higher than those of WT after drought treatment, while their anthocyanin contents and H_2_O_2_ accumulations were lower than those of WT (Figure S1D‐I). The temperature of the leaf surface is a phenotype that cannot be observed by the naked eye, but it could be investigated with infrared thermal imaging technology. This experiment revealed that LEA3‐35S‐OE and VOC‐35S‐OE plants achieved a stable state enabling their leaves to remain at a suitable temperature under drought stress (Figure S1D, G). To explore whether the differential sensitivities of the LEA3‐35S‐OE and VOC‐35S‐OE lines are associated with the expression levels of drought‐responsive marker genes, the relative expression levels of *RD29A*,* SnRK2.6* and *RbohD* were further determined by qRT‐PCR in WT and transgenic plants. The results showed that the expression levels of these genes in the OE lines were higher than those in WT (Figure S1F). *RD29A*,* RbohD* and *SnRK2.6* are drought responsive and have positive functions in drought tolerance (Ding *et al*., 2015). *RD29A* is a drought‐inducible gene with a promoter that contains two major cis‐acting elements, which are involved in stress‐inducible gene expression (Shinozaki and Yamaguchi‐Shinozaki, 2007). *RbohD* is involved in the stress response and ROS metabolic process, and it is required for hydrogen sulphide‐induced stomatal closure (Ding *et al*., 2015; He *et al*., 2018; Scuffi *et al*., 2018). SnRK2.6 is a protein kinase that functions in the gene regulatory pathway of ABA signal transduction and can be involved in plant drought tolerance through mediating phosphorylation (Ding *et al*., 2015; Kobayashi *et al*., 2005). Stomatal closure is a defensive action that is induced by ABA and accompanied by the production of H_2_O_2_ (He *et al*., 2018; Li *et al*., 2017; Zhang *et al*., 2001). The gene expression changes in the ABA pathway in the present study were consistent with this (Figures 6, 7, 8). OsbZIP23 was reported as a central regulator of ABA signalling and drought resistance in rice (Zong *et al*., 2016). Under drought stress, ZIP family gene expression and reduced H_2_O_2_ levels were identified in the BnLEA‐OE and VOC‐OE lines. These results support the hypothesis that ABA signal transduction could confer drought tolerance.

*LEA* genes are activated by DREBs (Battaglia *et al*., 2008; Lee *et al*., 2012; Morran *et al*., 2011). The expression of DREB in the differential LEA expression lines, as revealed by RNA‐seq analysis, was consistent with the known functions of LEA in *Arabidopsis*, wheat and barley (Morran *et al*., 2011; Sakuma *et al*., 2006). The products of LEA genes are often quite hydrophobic and are involved in the direct protection of the cell from stress by increasing membrane stability and processing proteins (Battaglia *et al*., 2008; Chakrabortee *et al*., 2007; Goyal *et al*., 2005; Tolleter *et al*., 2007). In transgenic *B. napus*, the membrane of BnLEA‐35S was more stable under drought conditions. In the differential *LEA* expression lines, AT2G39030 (N‐ACETYLTRANSFERASE ACTIVITY 1, NATA1) and AT5G24780 (VEGETATIVE STORAGE PROTEIN 1, ATVSP1) were the common DEGs in long‐term drought treatment (Figures 6d, S6). NATA1 acts as an ornithine N‐delta‐acetyltransferase, leading to the formation of N‐delta‐acetylornithine (Lou *et al*., 2016). ATVSP1 acts as an acid phosphatase similar to soya bean vegetative storage proteins, and it can be induced by wounding and jasmonic acid (Courteaux *et al*., 2013). These results indicate a gene expression trend similar to that of WTSL, but some key genes were still regulated in the late stage of drought stress in seeds. Many lipases were down‐regulated in the LEA‐OE lines. In addition, lipoxygenase genes were down‐regulated; for instance, AT3G45140 (*LOX2*) and AT1G72520 (*LOX4*) were down‐regulated in BnLEASS and AtLEASS, respectively. These results suggested that lipid degradation was down‐regulated in the seeds of the LEA‐OE lines. To further investigate transcript coexpression in the drought treatment, a K‐means clustering algorithm and network analysis were used to identify expressed transcripts with patterns of gene activity and to group genes with similar expression profiles (Figures S10, S11). These findings demonstrate that LEA can be active and play an important role in drought stress.

Methylglyoxal is a highly cytotoxic and mutagenic compound known to arrest growth (Singla‐Pareek *et al*., 2003). In plants, MG has been found to accumulate to higher levels under abiotic stress (Kaur *et al*., 2014). The glyoxalase pathway has been shown to be associated with abiotic stress adaptation, and GLY I and GLY II can function in salinity adaptation (Ghosh *et al*., 2014; Mustafiz *et al*., 2014; Singla‐Pareek *et al*., 2003). In *B. napus*, MG was significantly reduced by *BnVOC* in the drought treatment because of its enhancement of GLY I and GLY II activities (Figure 11). These results suggest that the glyoxalase system can function in drought stress and that increased expression of VOC can augment drought tolerance in *B. napus*.

In this study, the oil contents of the LEA‐OE and VOC‐OE lines were also increased. Microscopic observation of the seeds indicated that the *LEA* and *VOC* genes might have a function in enabling relatively normal seed development under drought stress. LEA proteins could bind to the cell membrane and maintain its integrity (Goyal *et al*., 2005). The results of microscopic observation in the present study show that LEA‐OE seeds had stable and thick membranes, which can maintain normal lipid synthesis in plant cells, especially under drought stress conditions. The complement of unsaturated FAs was found to be changed in the BnLEA‐OE and BnVOC‐OE lines compared with WT (Figure S12). The FA composition of transgenic plants subjected to drought was also changed (Figure S12), which indicated that overexpression of *BnLEA* and *BnVOC* could lead to changes in FA composition. LTPs are defined as proteins that facilitate/accelerate lipid exchange between membranes *in vitro* (D'Angelo *et al*., 2008). Our results showed that LTPs were down‐ and up‐regulated in the AtVOC‐RNAi and OE lines, respectively. This phenomenon could lead to more stable membrane structures under drought conditions and more efficient lipid fluxes in the OE lines. In the FA biosynthetic pathway, chloroplastic acetylcoenzyme A carboxylase 1 (CAC1), CAC2, transferase, 3‐ketoacyl‐acyl carrier protein synthase 1 (KAS1), an NAD(P)‐binding protein, hydroxyacyl‐[acyl carrier protein] dehydratase, MOD1, LACS9, and LACS1 were all up‐regulated in the seeds of the LEA‐OE lines (Figure 8). Notably, each step of the main FA biosynthetic pathway (CAC → AT2G30200 → KAS1 → AT1G24360 → AT5G10160 → MOD1 → LACS) had at least one up‐regulated gene in these OE lines (Figure S13). LACS can esterify free FAs to acyl‐CoAs, which is a key activation step that is necessary for the utilization of FAs by most lipid metabolic enzymes (Browse, 2002). The transcriptome and qPCR analyses of the hybrids showed that the expression of *LACS* was affected, suggesting that the final step of FA biosynthesis could be more efficient in the OE lines. Many LEA proteins localized to the cytosol and plastids, with most being able to diffuse into the nucleus. LEA are thus expected to establish interactions with various cellular membranes under stress conditions (Candat *et al*., 2014). The FAs were mainly synthesized in plastids, and membranes were important for cell stability in stress. On the other hand, events downstream of FA metabolism, especially FA degradation and lipid peroxidation, were down‐regulated. This reduced expression of genes related to FA degradation and lipid peroxidation also contributed to FA accumulation.

Reactive oxygen species usually act as major signalling molecules in plant hormone response pathways (Bright *et al*., 2006; Li *et al*., 2017). Among ROS, H_2_O_2_ is a versatile molecule that participates in the response to abiotic stress (Quan *et al*., 2008; Reczek and Chandel, 2015). High concentrations of H_2_O_2_ lead to cell injury (Dietz *et al*., 2016; Huang *et al*., 2016). In the present study, H_2_O_2_ contents were decreased in the OE lines and OE hybrids. Drought‐responsive genes contributed to these lower concentrations of H_2_O_2_, which could reduce cell injury in plants under drought stress. The content of MDA usually represents the level of lipid peroxidation. *VOC* genes might mitigate lipid peroxidation, which could also affect lipid production. Low concentrations of H_2_O_2_ and MDA were also found in other abiotic treatments in *B. napus* (Farooq *et al*., 2016; Nouairi *et al*., 2009). The activities of enzymes related to ROS metabolism (CAT, SOD) showed corresponding changes under drought stress (Figure 11). This result suggested that VOC could affect genes encoding the enzymes involved in ROS metabolism. MG and GSH are also involved in ROS metabolism, and the relative contents of these compounds were altered in the differential VOC expression lines of *B. napus* (Figure 10). The subcellular location of VOC was the nucleus, cell membrane, and cytosol (Figure S14), which indicate that they can function in MG and ROS metabolism. GLY I and GLY II can catalyse the MG detoxification with GSH (Singla‐Pareek *et al*., 2003). In the present study, the activities of the GLY I and GLY II enzymes and the contents of GSH in the differential VOC expression lines of transgenic *B. napus* indicated that VOC could function in MG metabolism. In addition, GLY I plays a key role in glyoxylate metabolism. The glyoxylate cycle could influence the final lipid accumulation in the plant glyoxysome via peroxidation. Under other abiotic stresses, glyoxylate metabolism in the seeds of different *B. napus* lines was also found to change (Zhou *et al*., 2018). The results of this study suggest that VOC plays a role in reducing ROS, which can lead to enhanced drought tolerance and lipid accumulation in *B. napus*.

Photosynthesis provides sucrose, which is the upstream substrate for FA biosynthesis (Durrett *et al*., 2008). Photosynthetic efficiency and photosynthesis‐related pathways were found to be up‐regulated in the OE transgenic lines and 35S hybrids of *B. napus* under drought stress. Previous studies have revealed that photosynthesis plays an important role in responses to abiotic stress (Garg *et al*., 2002; Ghosh *et al*., 2014; He *et al*., 2018; Munns and Tester, 2008). Under drought conditions, plants need to maintain their internal water balance and enable photosynthesis. In the present study, the transgenic *B. napus* lines maintained a balance between photosynthetic capability and osmotic loss by fine‐tuning stomatal closure, transpiration rate, chlorophyll content, RWC and photosynthetic rate (Figures 1, 8, S1, S7). The relative expression of photosystem genes was found to correspond to the expression level of the genes of interest. In addition, the chlorophyll contents of the OE lines were higher than those of the RNAi lines and WT. The changes in chlorophyll contents were consistent with the transcriptome analysis, in which the genes that were involved in chlorophyll synthesis were up‐regulated and down‐regulated in the OE and RNAi lines, respectively. For example, the important genes *CHLM*,* GUN5*,* CRD1* and *CHLG* were all up‐regulated in the LEA‐OE and VOC‐OE lines (Chen *et al*., 2016, 5; Lin *et al*., 2014; Schlicke *et al*., 2014; Van Wilder *et al*., 2009). This maintenance of chlorophyll synthesis in the leaves of the OE lines could contribute to the enhanced photosynthesis in the drought treatment. The higher carbon fixation levels and elevated capacity for photosynthesis also contribute to FA accumulation. Previously, an integrated omics analysis of *B. napus* and *Glycine max* also showed that some genes involved in photosynthesis were related to lipid metabolism (Zhang *et al*., 2018). Thus, the enhanced photosynthetic efficiency in the LEA‐OE and VOC‐OE lines could confer drought tolerance and contribute to lipid accumulation.

In summary, we functionally identified different copies of drought response genes from *B. napus* and *Arabidopsis*. The *LEA* and *VOC* genes could contribute to drought tolerance and oil content in both *B. napus* and *Arabidopsis*. Hybridization of lines overexpressing these two genes showed reinforced performance in drought tolerance and oil production. The mechanism by which LEA and VOC improve oil accumulation is mainly by enhancing photosynthetic efficiency and reducing ROS under drought stress. Therefore, these drought response genes could not only function in drought stress but also improve oil accumulation. The present results provide novel insights into the relationship between oil accumulation and drought resistance and provide a new way to improve oil production in the breeding of oil crops.

## Experimental procedures

### Plant materials and growth conditions

The WT *Arabidopsis* used was Columbia ecotype, and *B. napu*s cv. Westar was used as WT *B. napus*. The T‐DNA insertion mutant of the *atlea3* gene was obtained from the Arabidopsis Biological Resource Center and then identified by PCR. *Arabidopsis* was planted, and the growth conditions were established according to a previous study (Zhang *et al*., 2013). The *B. napus* seeds were similarly sterilized and grown under the same conditions. After growing for 4 weeks, the *B. napus* plants were transplanted to fields until harvest.

### Vector constructs and plant transformation

Four gene copies of *BnLEA3* and *BnVOC* from *B. napus* were acquired and named BnLEA3.1, BnLEA3.2, BnLEA3.3, BnLEA3.4, BnVOC1, BnVOC2, BnVOC3, and BnVOC4 (GenBank Nos. KP315905, KP315906, KP315907, KP315908, KP315909, KP315910, KP315911, KP315912), respectively. These copies were selected because their expression levels were relatively stable and higher in seeds and leaves during drought, based on our previous studies (Liang *et al*., 2016, 2017). In addition, they were located in four different A or C subgenomes of *B. napus*. The homologous *AtLEA3* and *AtVOC* genes (AT1G02820, AT1G07645) in *Arabidopsis* were cloned. The pGEX and pCAMBIA1305 vectors were used for prokaryotic expression and for functional complementation in the *atlea3* mutant, respectively. These plasmids were transformed into *Arabidopsis* and *B. napus* using Agrobacterium GV3101 with the floral dip method and the *B. napus* transformation method (Cardoza and Stewart, 2003). The DsRed marker was used for the selection of transformant seeds, which were also PCR confirmed (Zhang *et al*., 2013).

### RNA isolation and RT‐qPCR analysis

Gene‐specific primers were designed using Primer 5.0 (Table S2). A housekeeping gene (*ACTIN*) that is constitutively expressed in *B. napus* and *Arabidopsis* was used as a reference for normalization and analysed using three biological replicates for the qPCR analysis. RNA isolation and RT‐qPCR analysis were performed according to a previous study (Liang *et al*., 2016). Twenty DEGs that were observed in FA biosynthesis, photosynthesis, ROS metabolism and other random genes were selected for RT‐qPCR. As shown in Figure S11, the RT‐qPCR results generally matched the RNA‐seq results.

### Generation of transgenic plants and confirmation through southern hybridization and Western blotting

The presence of the transgene and its expression were first confirmed by RT‐qPCR and then by Southern blotting and Western blotting. For Southern blotting, genomic DNA that tested positive by PCR was digested by the *Hind*III enzyme, blotted and probed by using the hygromycin gene following the standard protocol (Mustafiz *et al*., 2014). For Western blotting, FLAG was used as the tag, and ACTIN was used as the housekeeping protein. The next steps were performed according to the protocol (Lagarde *et al*., 1996).

### Drought treatment of the experimental materials

In *Arabidopsis*, 15% PEG for 9–10 h and withholding water for 10–15 days (RWC of soil was <40%) were used for the short‐term and long‐term drought treatments, respectively. The leaf samples were collected from plants that were 4 weeks (short‐term) and 5 weeks (long‐term) after germination. The silique samples were collected from plants 6 weeks (short‐term) and 7 weeks (long‐term) after flowering. The drought response marker genes (*SnRK2.6, RbohD* and *RbohF*) started to be up‐regulated in short‐term drought and reached peak levels in long‐term drought (Tran *et al*., 2004; Zheng *et al*., 2016). In *B. napus*, water was withheld from the plants 10 days after flowering for 15 days.

### 3,3′‐Diaminobenzidine (DAB) staining and anthocyanin measurements

DAB staining was performed as described previously (He *et al*., 2018), and the anthocyanin measurements were conducted according to a previous report (Gou *et al*., 2011).

### Morphological observation and analysis

For SEM, TEM and light observation, the samples were fixed and prepared according to the previously published methods (Dolan *et al*., 1993; Gan *et al*., 2013; Girard *et al*., 2017) and observed using a scanning electron microscope (Hitachi S‐4700, Japan), a transmission electron microscope (Hitachi t7700, Japan), and a light microscope (Olympus BX53, Japan), respectively. The leaf surface temperature images were taken using a Thermal Imager (Fluke Ti400) and analysed using Fluke SmartView Infrared Analysis Software. The seed sizes were scanned and measured by a crop scanning test system (Wanshen SC‐G, China).

### Oil content and FA analysis of experimental materials

The lipids of the seeds were extracted by the protocol described in a previous study, and the oil contents were measured as the proportion of total oil content to seed dry weight. The change percentages were measured as change percentage = (oil content of sample‐oil content of CK)/oil content of CK *100 (Maisonneuve *et al*., 2010; Zhang *et al*., 2013). The extracted FAs were analysed by GC/MS using an Agilent 6850 gas chromatograph with frame ionization detection (FID) on a polar column (Agilent, DB‐23, 30 m by 0.25 mm i.d., 0.25 μm film).

### Library generation for high‐throughput sequencing and bioinformatics analyses

Method S1 shows details of the RNA sequencing and bioinformatics analyses (Figures [Link], [Link], [Link], [Link], [Link], [Link], Tables S3 and S4).

### Measurement of MG, H_2_O_2_, MDA, GSSG and GSH concentrations

The contents of MG, GSH, GSH, H_2_O_2_ and MDA were measured using a plant MG ELISA kit (Nanjing Jin Yibai, JEB~14422, China), a Reduced Glutathione (GSH) Assay Kit (Solarbio BC1170, China), an Oxidized Glutathione (GSSG) Assay Kit (Solarbio BC1180, China) or according to Shi's and Basu's studies (Basu *et al*., 2001; Shi *et al*., 2014;).

### Enzyme assays

For the enzyme assays, the plant samples were prepared and quantified using previously published protocols (Doderer *et al*., 1992; Shi *et al*., 2014; Singla‐Pareek *et al*., 2003).

### Measurement of photosynthetic parameters

An infrared gas analyser (Li‐COR 6400, Agilent, Lincoln, Nebraska, USA) was used to detect photosynthetic parameters.

## Conflict of interest

The authors declare no conflict of interest.

## Author contributions

ML designed the research and wrote the article. YL and LG performed the vector constructions, transformation, GCMS, and data analysis and wrote the article. KK, YL and JG conducted bioinformatics analyses and discussed the results. SN, JX, SS, LX and SL performed the crossing, fieldwork and oil extractions. LG and YY conducted the drought treatment. JX, SL and BW performed the enzyme assays and MDA, MG, H2O2, GSSG and GSH and measurements.

## Supporting information


**Figure S1** Enhanced drought tolerance in LEA3‐OE and VOC‐OE lines of Arabidopsis.

**Figure S2** Identification of mutant *atlea3* and gene expression of transgenic Arabidopsis.

**Figure S3** Oil contents of the *atlea3* mutant and HB lines.

**Figure S4** qPCR of *LEA* and *VOC* genes in *B. napus*.

**Figure S5** Oil reduction comparison.

**Figure S6** FA compositions of OE, RNAi and hybrid seeds in *B. napus*.

**Figure S7** FA compositions of hybrid *Arabidopsis*.

**Figure S8** Prokaryotic expression of VOC genes in *E. coli*.

**Figure S9** Microscopic observations of OE and WT seeds and leaves in *Arabidopsis* under drought conditions.

**Figure S10** K‐means clusters and RT‐qPCR confirmation of DEGs.

**Figure S11** Network of DEGs in LEA and VOC transgenic seeds under drought conditions.

**Figure S12** FA compositions of OE and WT seeds in *Arabidopsis*.

**Figure S13** Change folds of gene in the FA synthesis in LEA‐relative lines.

**Figure S14** Subcellular localization of VOC.

**Table S1** Detailed oil and FA contents of transgenic *Arabidopsis* overexpressing *LEA* and *VOC*.

**Table S2** Primers used in RNA‐seq confirmation

**Table S3** Detailed RNA‐seq information.

**Table S4** Abbreviations used in this study.

**Method S1** RNA‐seq analysis methods.
