# Supplementary figures and images for: Drought‐responsive genes, late embryogenesis abundant group3 ( LEA3) and vicinal oxygen chelate, function in lipid accumulation in Brassica napus and Arabidopsis mainly via enhancing photosynthetic efficiency and reducing ROS
Source: Plant Biotechnol J. 2019 Apr 26;17(11):2123–42. doi: 10.1111/pbi.13127 (PMC6790364; doi:10.1111/pbi.13127)

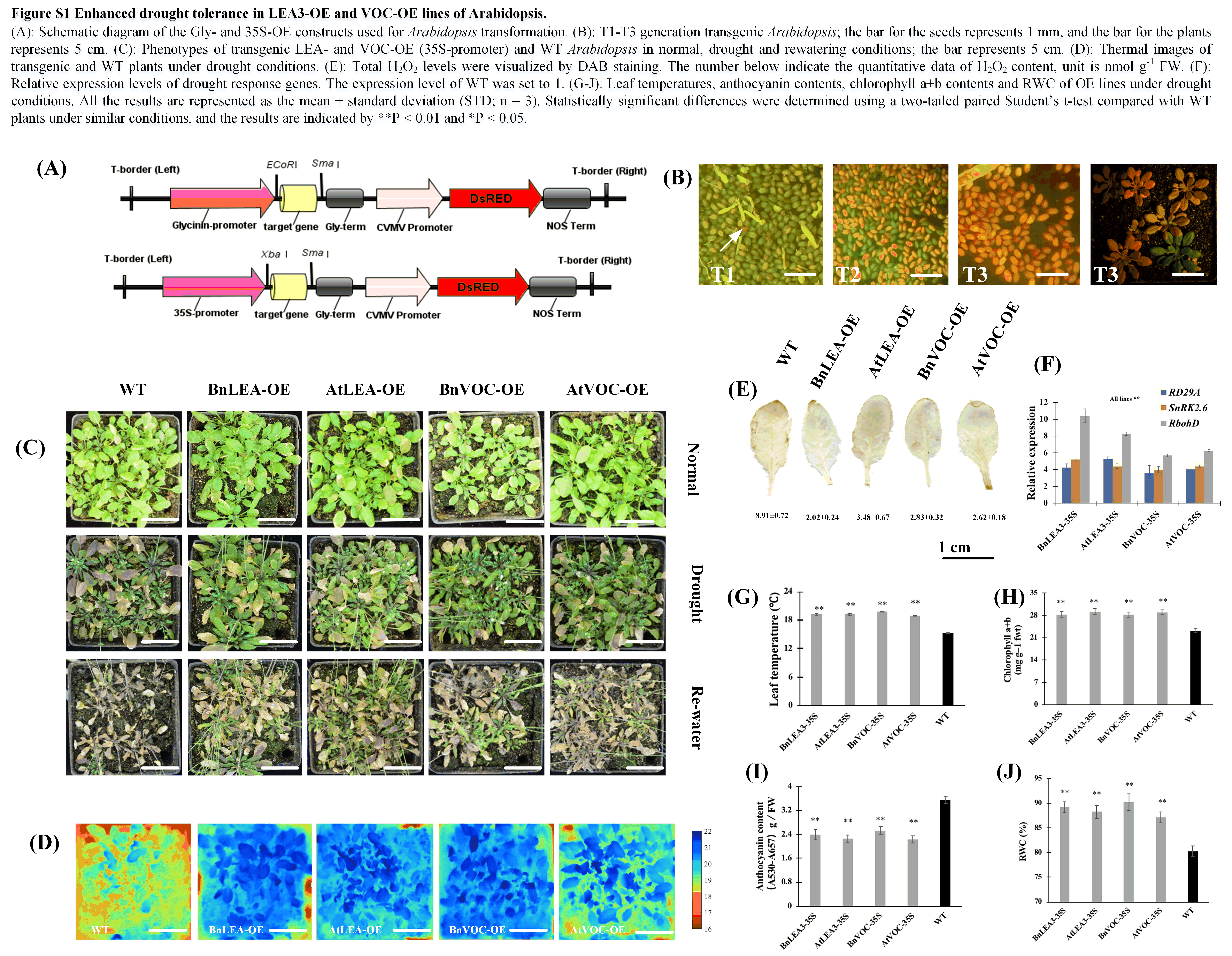

Supplement: Supplementary file 1 — Figure S1 Enhanced drought tolerance in LEA3‐OE and VOC‐OE lines of Arabidopsis. [file PBI-17-2123-s019.png]

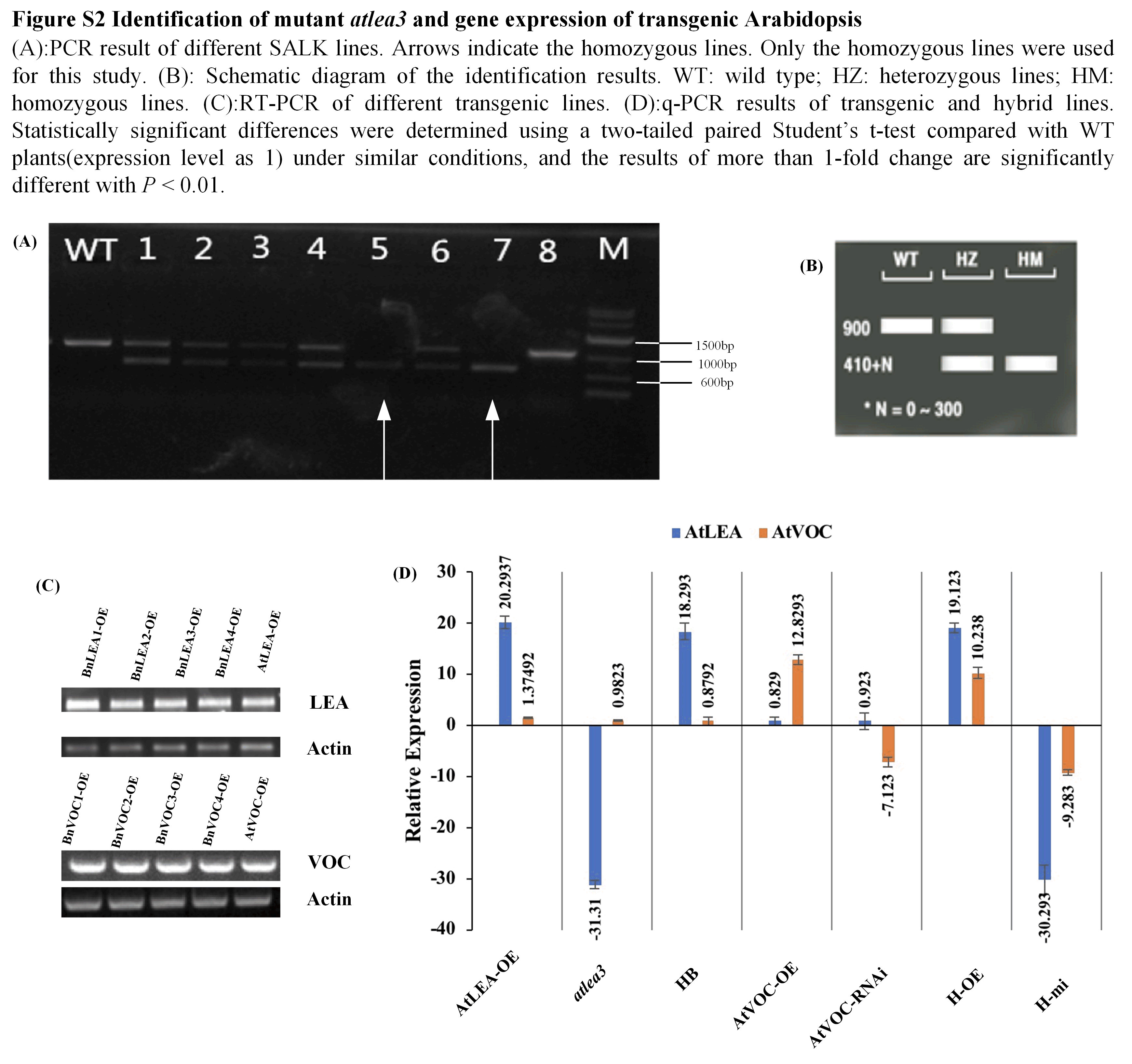

Supplement: Supplementary file 2 — Figure S2 Identification of mutant atlea3 and gene expression of transgenic Arabidopsis. [file PBI-17-2123-s001.png]

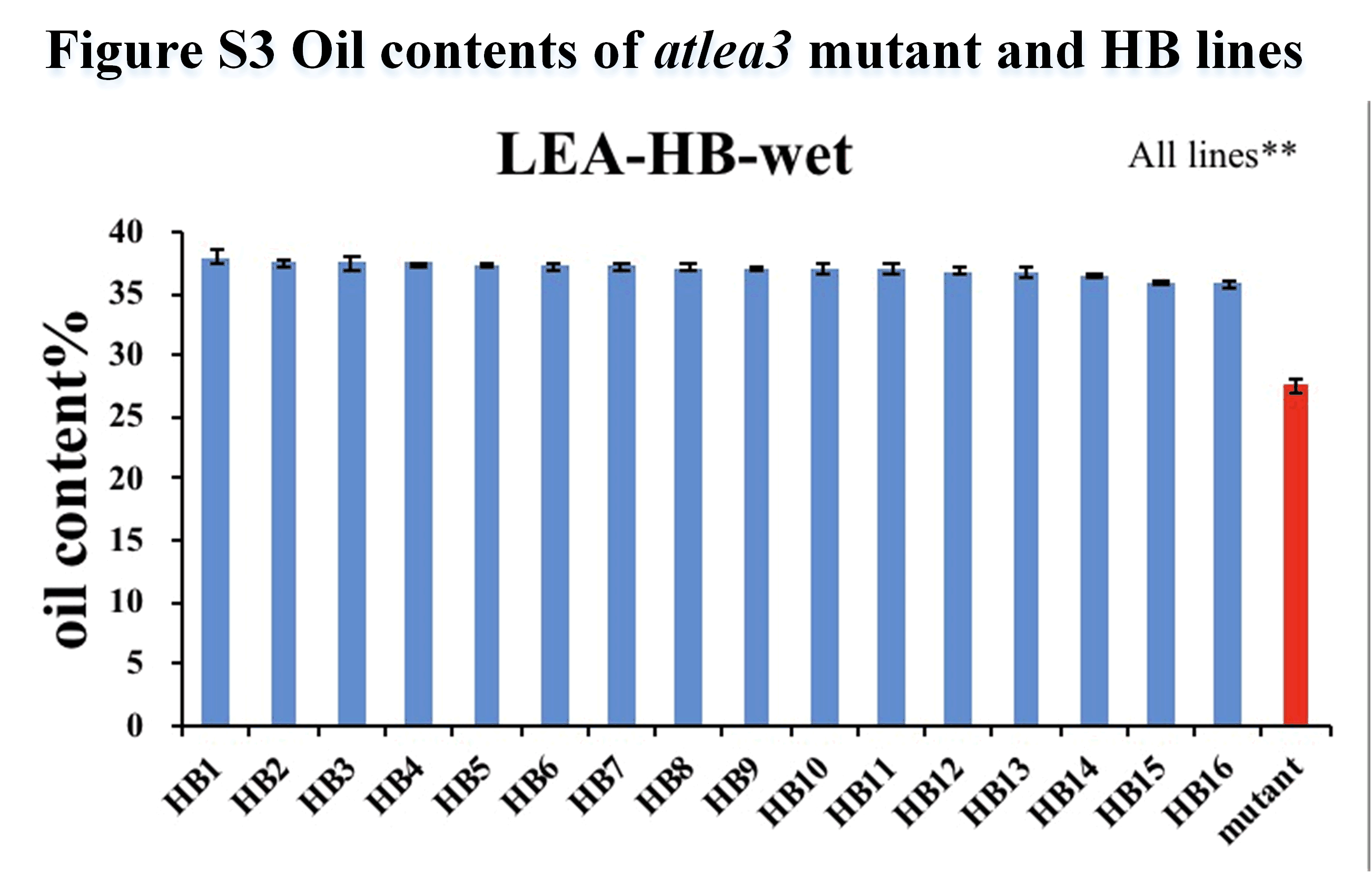

Supplement: Supplementary file 3 — Figure S3 Oil contents of the atlea3 mutant and HB lines. [file PBI-17-2123-s002.png]

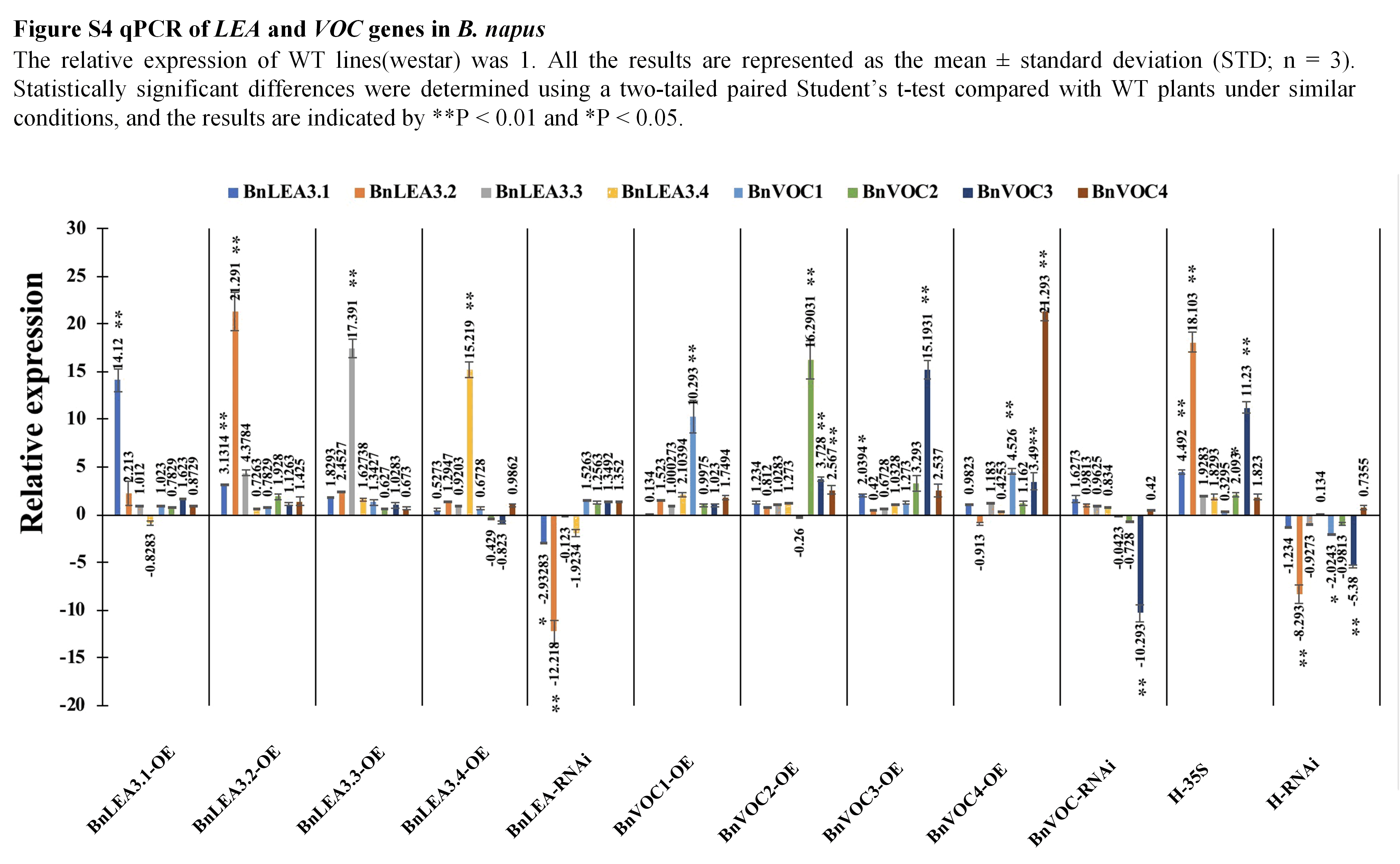

Supplement: Supplementary file 4 — Figure S4 qPCR of LEA and VOC genes in B. napus. [file PBI-17-2123-s003.png]

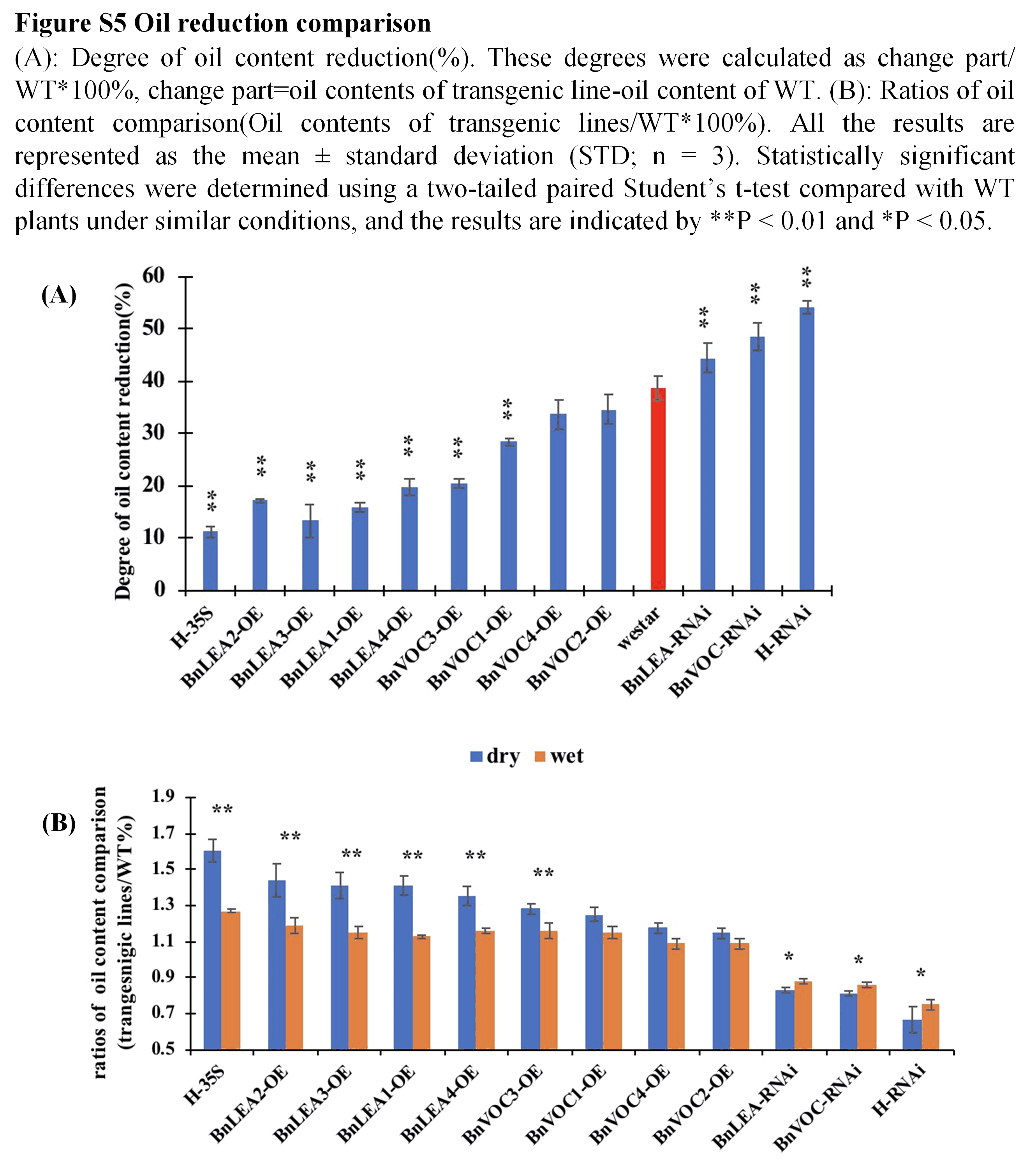

Supplement: Supplementary file 5 — Figure S5 Oil reduction comparison. [file PBI-17-2123-s005.png]

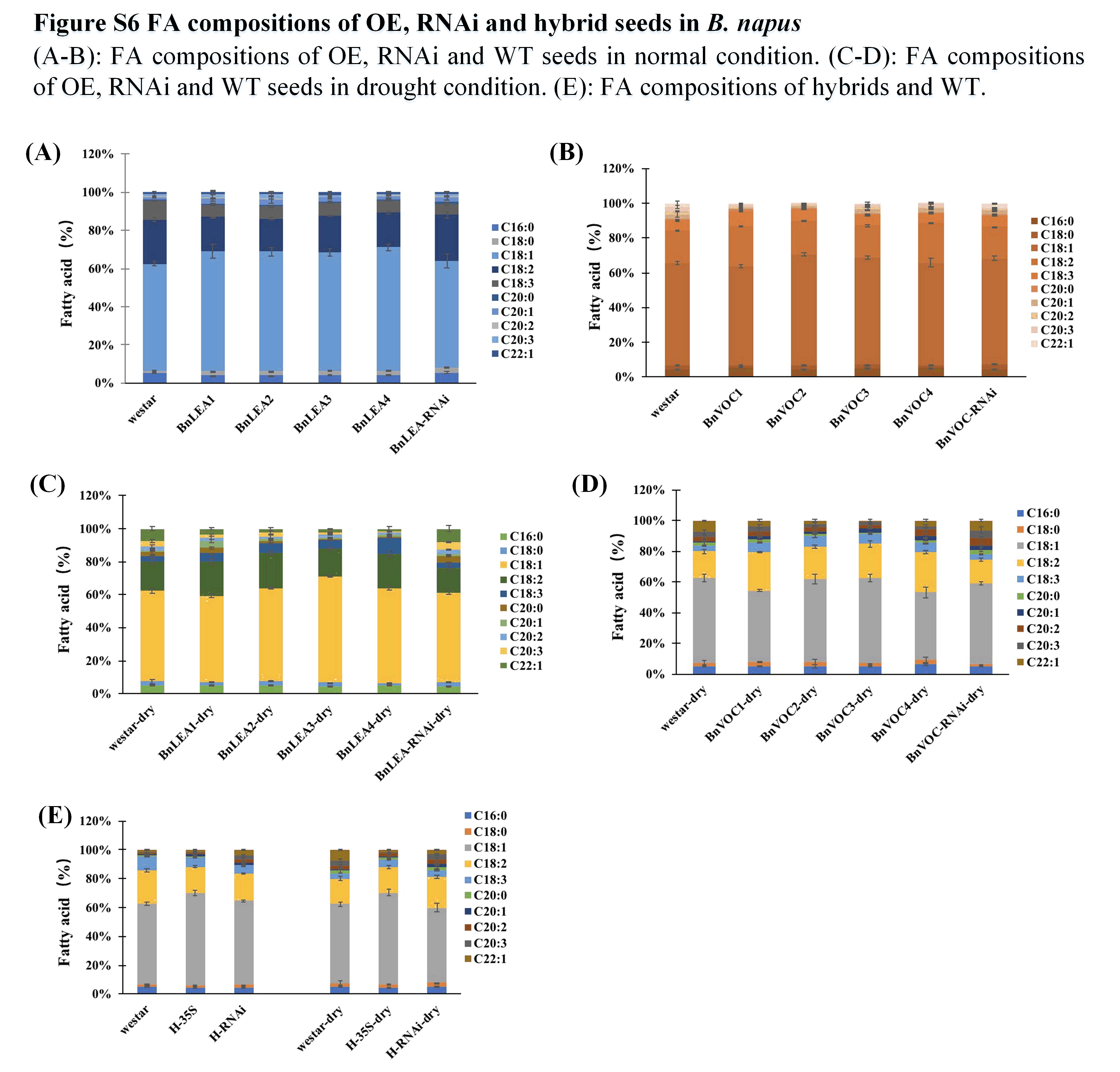

Supplement: Supplementary file 6 — Figure S6 FA compositions of OE, RNAi and hybrid seeds in B. napus. [file PBI-17-2123-s006.png]

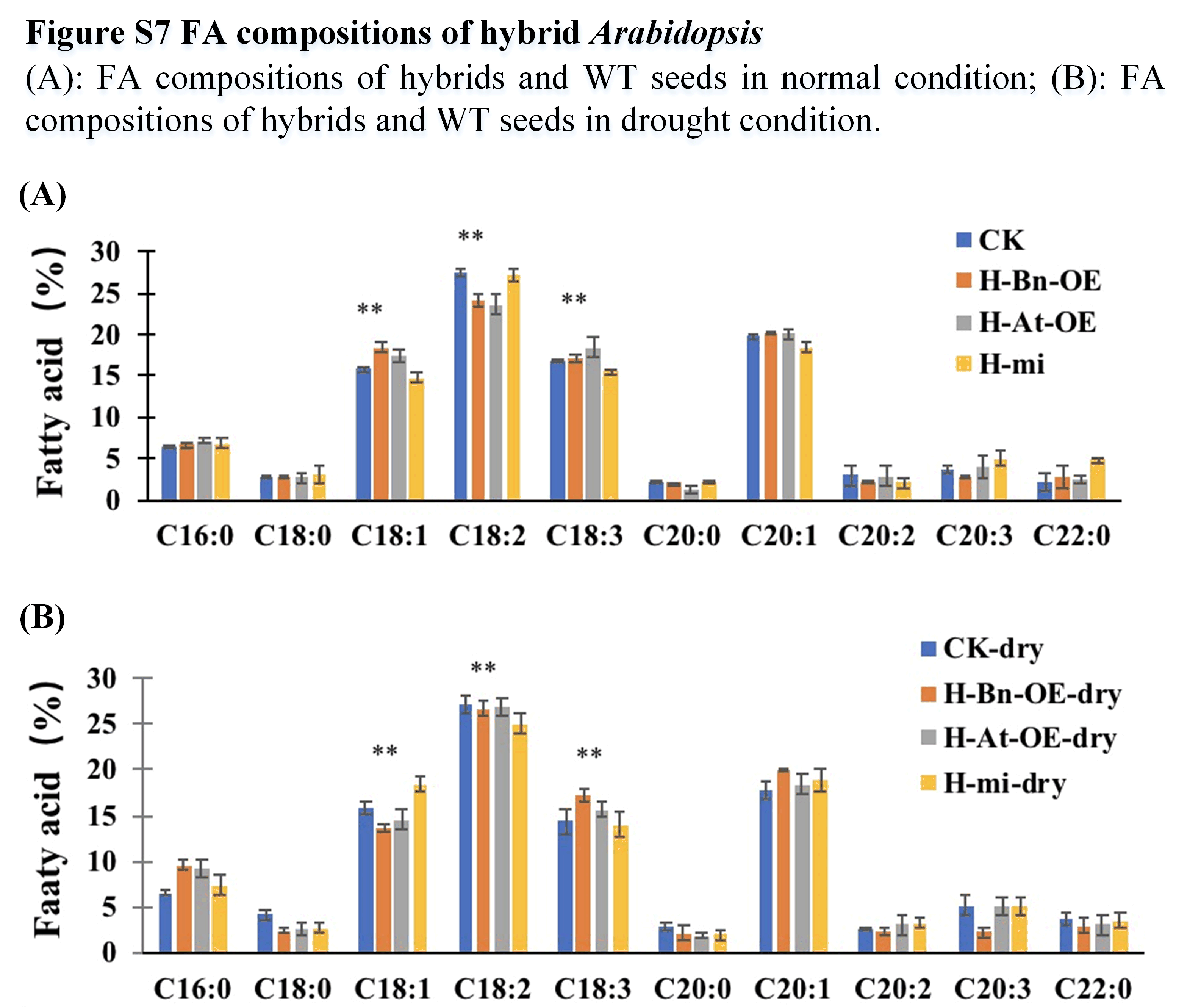

Supplement: Supplementary file 7 — Figure S7 FA compositions of hybrid Arabidopsis. [file PBI-17-2123-s007.png]

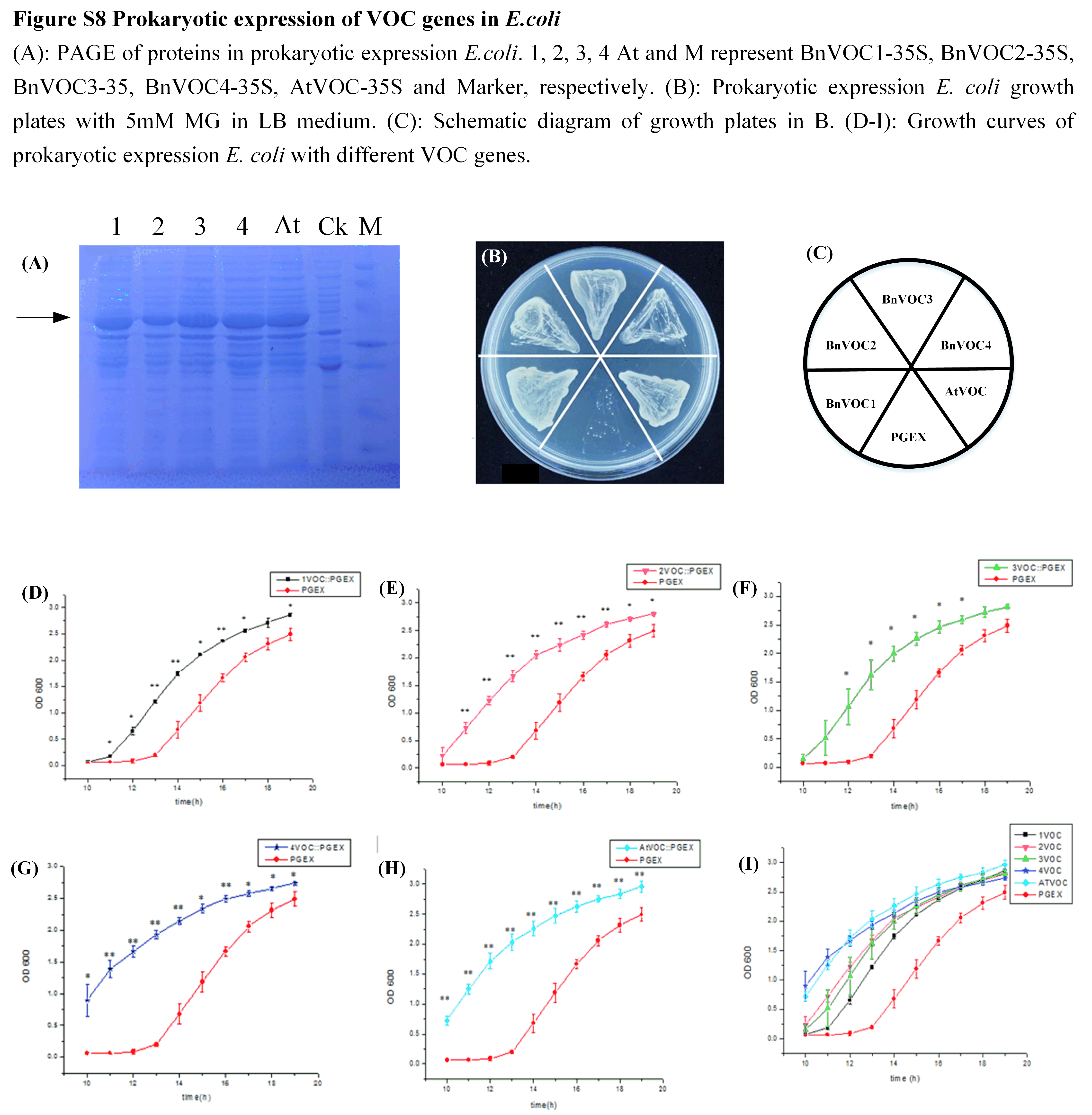

Supplement: Supplementary file 8 — Figure S8 Prokaryotic expression of VOC genes in E. coli. [file PBI-17-2123-s004.png]

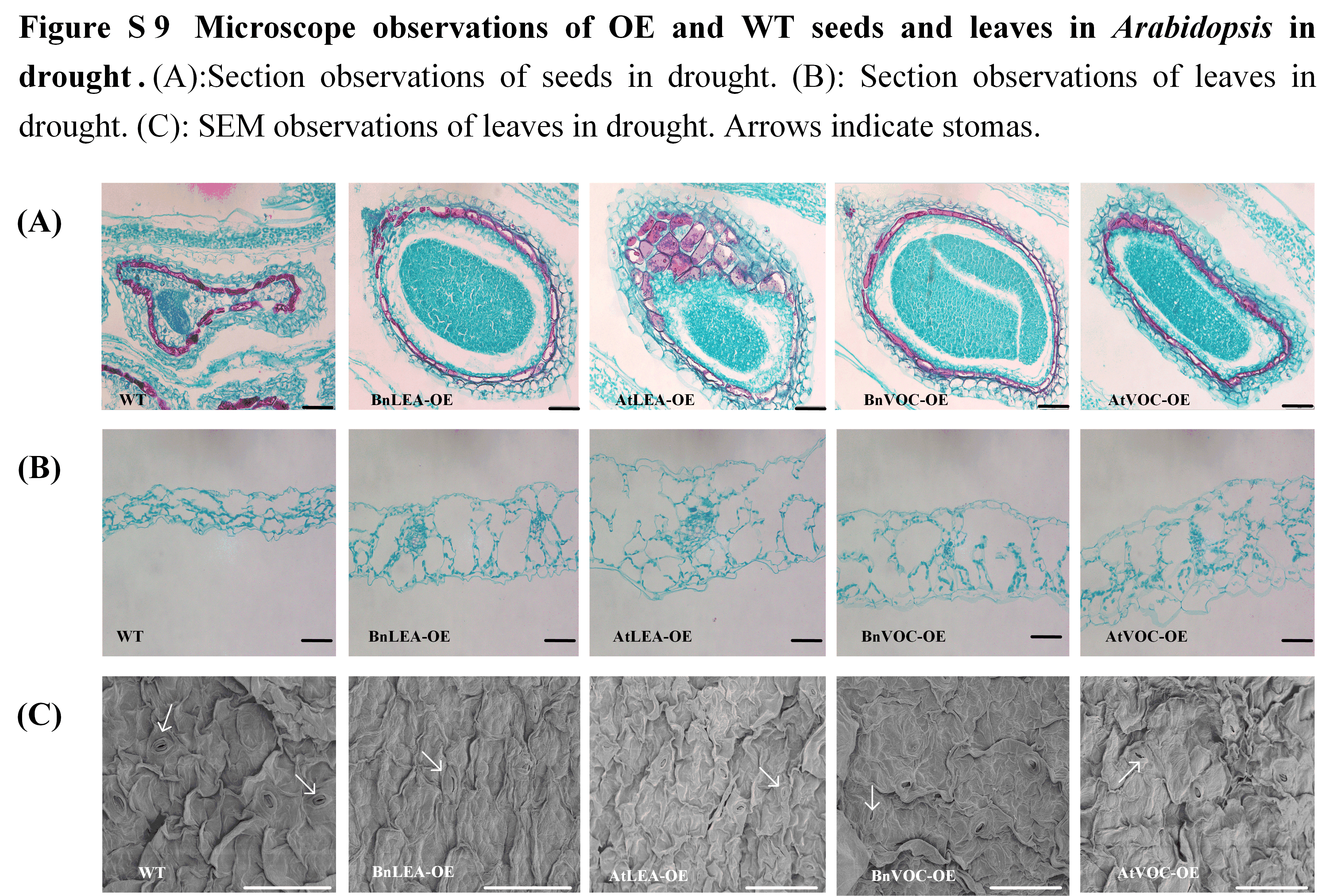

Supplement: Supplementary file 9 — Figure S9 Microscopic observations of OE and WT seeds and leaves in Arabidopsis under drought conditions. [file PBI-17-2123-s008.png]

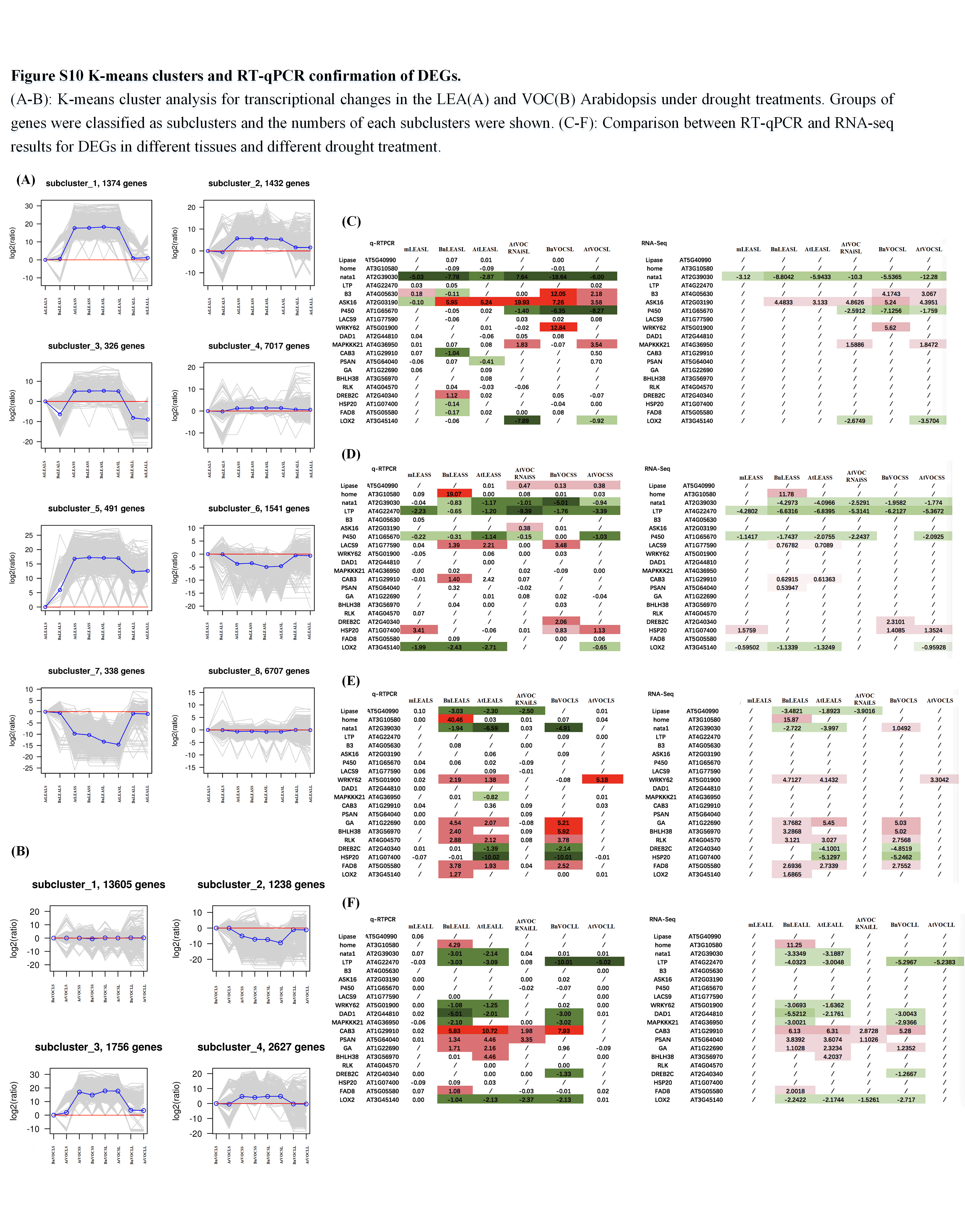

Supplement: Supplementary file 10 — Figure S10 K‐means clusters and RT‐qPCR confirmation of DEGs. [file PBI-17-2123-s009.png]

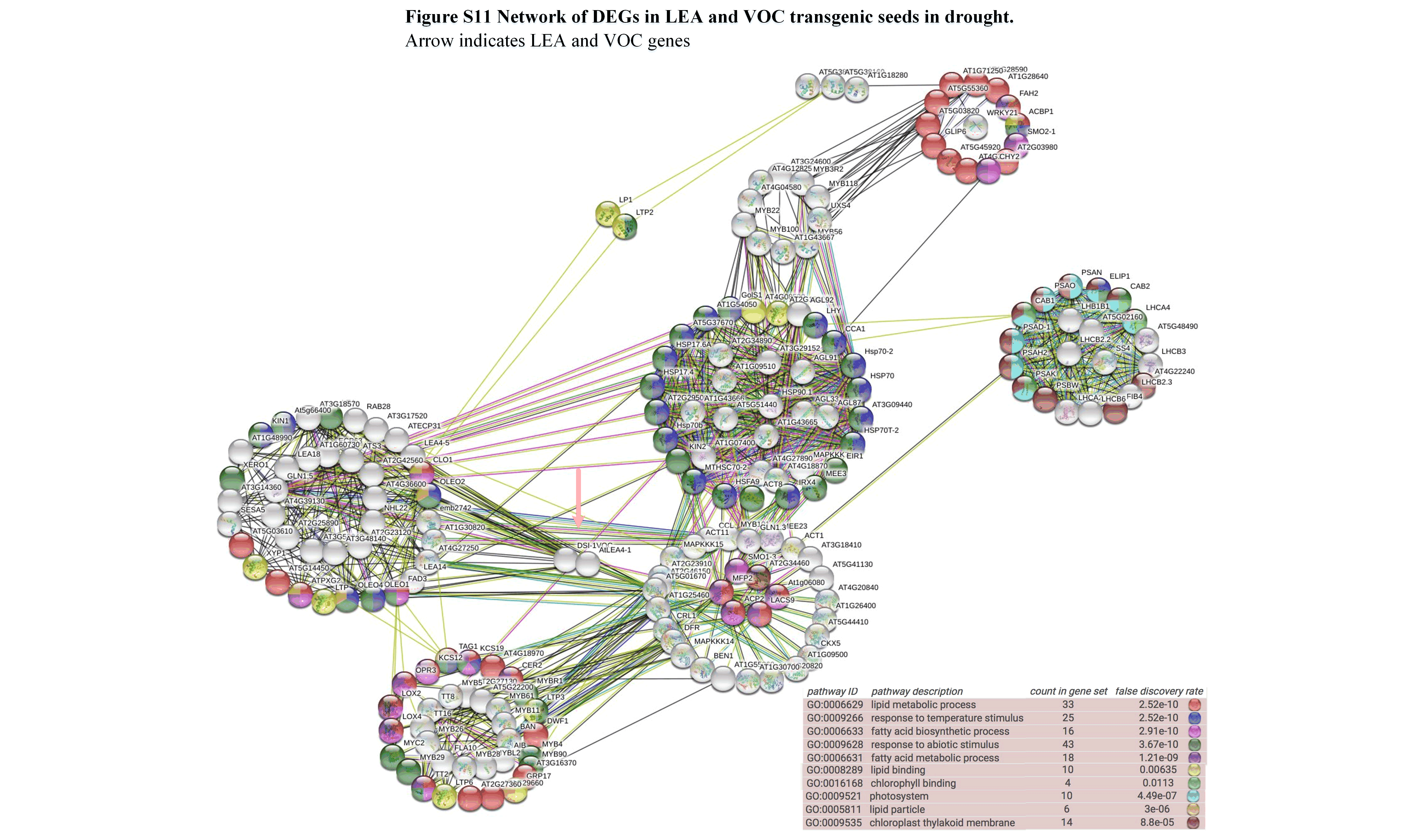

Supplement: Supplementary file 11 — Figure S11 Network of DEGs in LEA and VOC transgenic seeds under drought conditions. [file PBI-17-2123-s010.png]

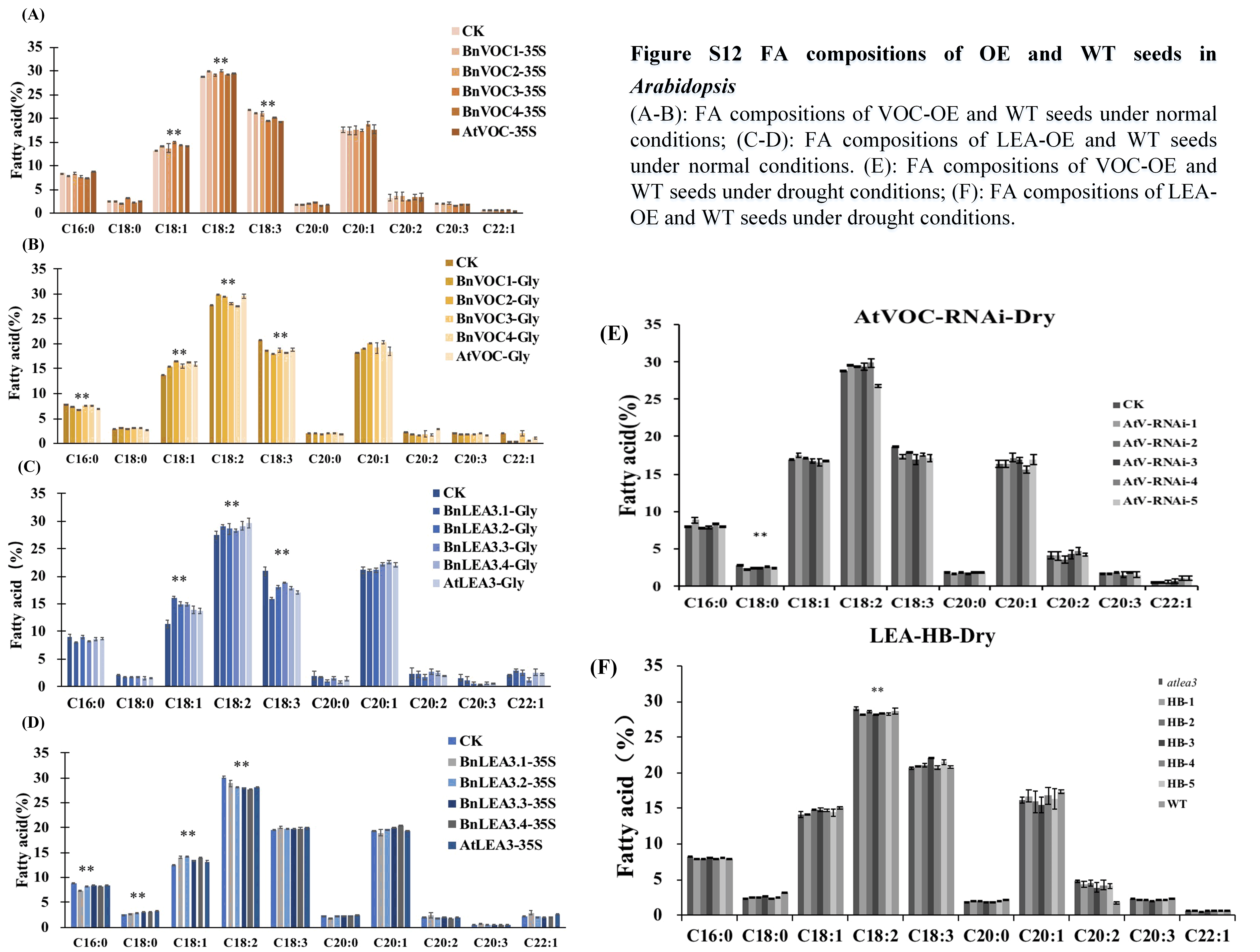

Supplement: Supplementary file 12 — Figure S12 FA compositions of OE and WT seeds in Arabidopsis. [file PBI-17-2123-s011.png]

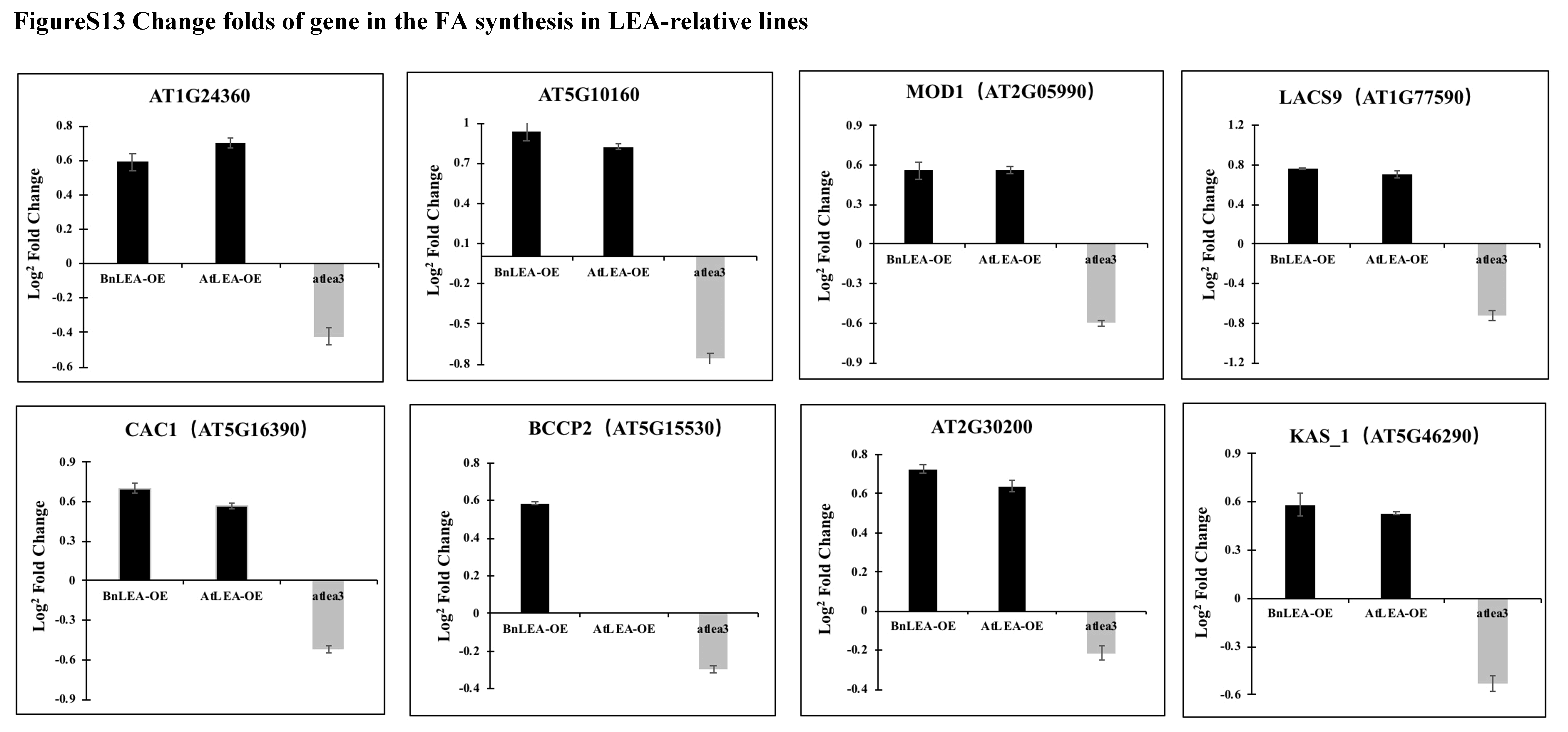

Supplement: Supplementary file 13 — Figure S13 Change folds of gene in the FA synthesis in LEA‐relative lines. [file PBI-17-2123-s017.png]

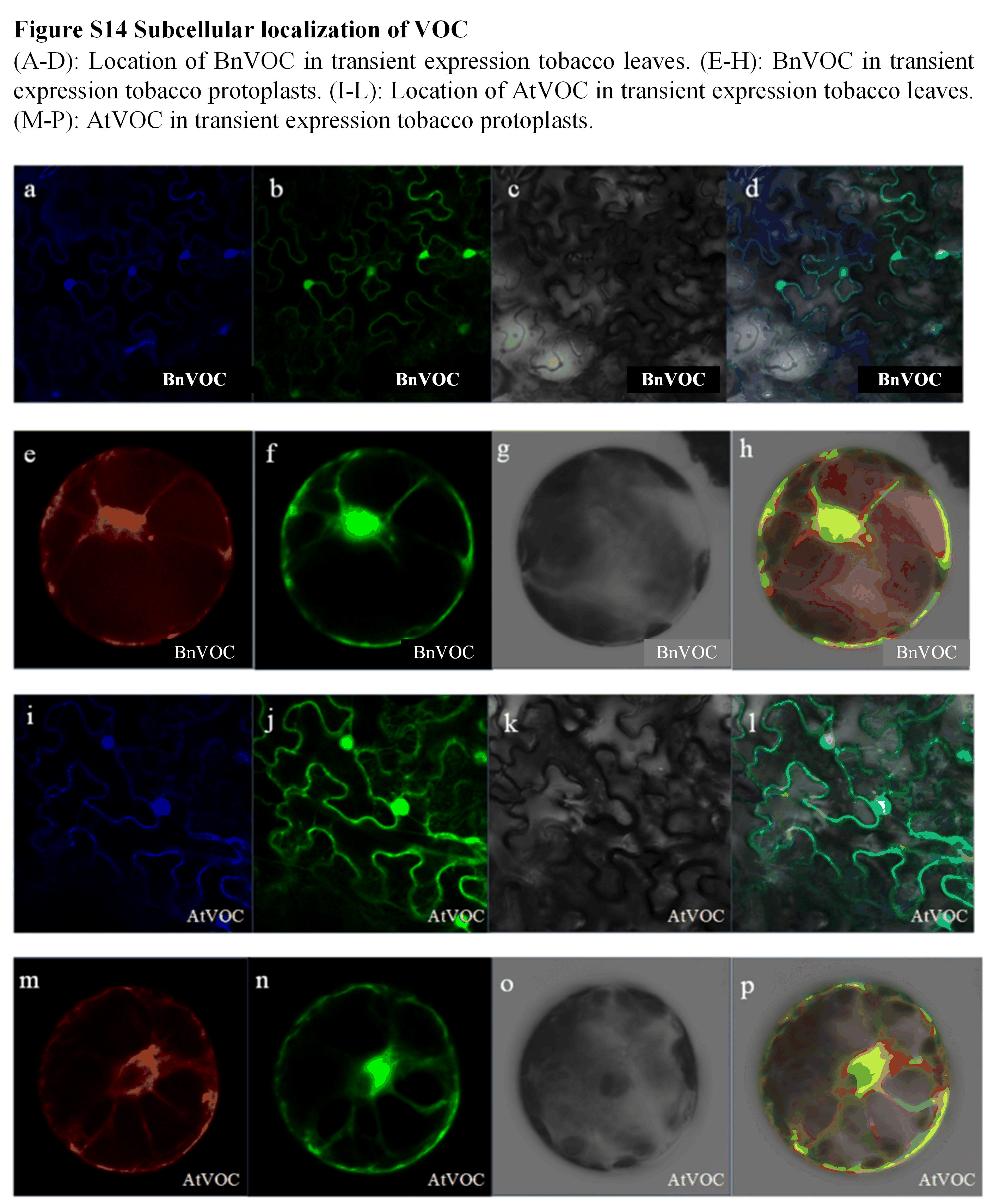

Supplement: Supplementary file 14 — Figure S14 Subcellular localization of VOC. [file PBI-17-2123-s016.png]
