# Supplementary material for: Drought‐responsive genes, late embryogenesis abundant group3 ( LEA3) and vicinal oxygen chelate, function in lipid accumulation in Brassica napus and Arabidopsis mainly via enhancing photosynthetic efficiency and reducing ROS
Source: Plant Biotechnol J. 2019 Apr 26;17(11):2123–42. doi: 10.1111/pbi.13127 (PMC6790364; doi:10.1111/pbi.13127)
Supplement: Supplementary file 19 — Method S1 RNA‐seq analysis methods. [file PBI-17-2123-s018.docx]

# Supporting information methods

**Library generation for mRNA sequencing**

Total RNAs were isolated from 56 samples (Accession No in TableS3) using TRIzol (Invitrogen, USA) according to the manufacturer’s instructions, and were treated with RNase-free DNase I (Promega) to remove contaminated residual genomic DNA. mRNAs were extracted from the total RNAs using Dynabeadsoligo (dT) (Invitrogen Dynal). First- and second-strand cDNAs were generated using SuperScripts II reverse transcriptase (Invitrogen) and random hexamers. Double-stranded cDNAs were fragmented by nebulization and sequencing libraries were prepared following the IIIumina RNA Seq library kit protocol (New England BioLabs Inc., Ipswich, USA) and were sequenced by the Illumina Hiseq X TEN platform (Illumina, USA). Quality control was performed by deleting the reads that contained >5% N^1^. Two biological replicates were used.

**Bioinformatics Analyses**

**Blasting clean reads to the reference genome**

Clean RNA-seq reads were mapped against the reference genome assemblies of Arabidopsis (*Arabidopsis thaliana*) ^2^ by SOAPaligner/SOAP2 software. We used homology-based methods to characterize the function of the genes annotated in the *A.* *thaliana* genome. The gene expression level was quantified using the same reference genome. The quality control of the nucleotide mismatches was less than five bases. The quality of the RNA-seq experiment depends on the read coverage with respect to the reference genome of Arabidopsis. A transcript can be considered as ’detected’ if the Fragments Per Kilobase of gene per Million reads mapped (FPKM) level was≥1 in plant tissue^3^. Transcripts were further divided into four levels, with low (1≤FPKM<15), moderate (3≤FPKM<15), or high (15≤FPKM<60), extremely high (FPKM≥60) accumulation levels (Figure 8; Figure S1; Table S3)

**Identification of gene expression levels and DEGs**

Gene expression levels represented by expected number of Fragments Per Kilo base of transcript sequence per Millions base pairs sequenced (FPKM)^4,5^. Differentially expressed genes (DEGs) between treatments were screened using the R package DESeq2^6^. The threshold value was set by |log2ratio|≥1 and false discovery rate (FDR)≦0.01. Genes with significance values of p ≤ 0.05 were considered to be non-additively expressed

**K-means clustering**

K-means clustering was performed by the ‘kmeans’ function in R package ‘stats’ (v. 3.2.2), where K value refers to the number of clusters grouped in the data set. The genes used here is the union of DEGs. The algorithm randomly assigned each gene into one of subclusters and located the centroid of each cluster according to the average FPKM value in two replicates. The data were normalized as log_2_^(FPKM+1)^.

## GO enrichment analysis

Gene Ontology (GO) annotation was performed using he GOseq R package in which gene length bias was corrected. Besides, AgriGO (a Web-based tool and database for gene ontology analysis; http://bioinfo.cau.edu.cn/ agriGO/) was used to analyze in the present study^7^. The GO terms with an adjusted p-value of ≤ 0.05 were defined as significantly enriched GO terms in DEGs.

**KEGG enrichment analysis**

Kyoto Encyclopedia of Genes and Genomes (KEGG) pathways annotation was performed using BLAST with an E-value cut-off of 1E-05 against the KEGG database^8^. Enrichment analysis was conducted by the software path-finder with a q-value 0.05 as the confidence level. The enrich level of KEGG enrichment is measured by the rich factor, *q*-value, and the number of genes enriched in the pathway using the whole expressed genes as background^9^.

**Analysis of Transcription Factors**

The transcription factors of DEGs were used to analyze network in the Plant Transcription Factor Database. Genome-wide regulatory interactions curated from literature and inferred by combining TF binding motifs and regulatory elements were performed for analyze. The maps were obtain from PlantRegMap^10^.

**Network Analysis of Protein-Protein interactions**

The proteins encoded by DEGs were used to analyze their interactions. All these proteins were performed in String database with the parameters: evalue=1e^-10^; max_target_seqs=1^11^. The PPi score were shown in DataS. The Cytoscape-V3.2.0 with the Agilent Literature Search Plug-in was also used in this study for visualization of network^11^.

**References**

1. Wang, X. *et al.* Genome-Wide and Organ-Specific Landscapes of Epigenetic Modifications and Their Relationships to mRNA and Small RNA Transcriptomes in Maize. *Plant Cell* **21,** 1053 (2009).

2. The Arabidopsis Genome Initiative. Analysis of the genome sequence of the flowering plant Arabidopsis thaliana. *Nature* **408,** 796 (2000).

3. Chan, A. C. *et al.* Tissue-specific laser microdissection of theBrassica napusfuniculus improves gene discovery and spatial identification of biological processes. *Journal of Experimental Botany* **67,** 3561–3571 (2016).

4. Trapnell, C. *et al.* Transcript assembly and quantification by RNA-Seq reveals unannotated transcripts and isoform switching during cell differentiation. *Nature Biotechnology* **28,** 511 (2010).

5. Belmonte, M. F. *et al.* Comprehensive developmental profiles of gene activity in regions and subregions of the <em>Arabidopsis</em> seed. *Proc Natl Acad Sci USA* **110,** E435 (2013).

6. Love, M. I., Huber, W. & Anders, S. Moderated estimation of fold change and dispersion for RNA-seq data with DESeq2. *Genome Biology* **15,** 550 (2014).

7. Du, Z., Zhou, X., Ling, Y., Zhang, Z. & Su, Z. agriGO: a GO analysis toolkit for the agricultural community. *Nucleic Acids Research* **38,** W64–W70 (2010).

8. Guimerà, R. & Nunes Amaral, L. A. Functional cartography of complex metabolic networks. *Nature* **433,** 895 (2005).

9. Mortazavi, A., Williams, B. A., McCue, K., Schaeffer, L. & Wold, B. Mapping and quantifying mammalian transcriptomes by RNA-Seq. *Nature Methods* **5,** 621 (2008).

10. Jin, J. *et al.* PlantTFDB 4.0: toward a central hub for transcription factors and regulatory interactions in plants. *Nucleic Acids Research* **45,** D1040–D1045 (2017).

11. Szklarczyk, D. *et al.* The STRING database in 2011: functional interaction networks of proteins, globally integrated and scored. *Nucleic Acids Research* **39,** D561–D568 (2011).
